# Tomato facultative parthenocarpy results from Sl *AGAMOUS‐LIKE 6* loss of function

**DOI:** 10.1111/pbi.12662

**Published:** 2016-12-27

**Authors:** Chen Klap, Ester Yeshayahou, Anthony M. Bolger, Tzahi Arazi, Suresh K. Gupta, Sara Shabtai, Björn Usadel, Yehiam Salts, Rivka Barg

**Affiliations:** ^1^ The Institute of Plant Sciences The Volcani Center Agricultural Research Organization Rishon LeZion Israel; ^2^ Institut für Biologie I RWTH Aachen Aachen Germany; ^3^ Institut für Bio‐und Geowissenschaften 2 (IBG‐2) Plant Sciences Forschungszentrum Jülich Jülich Germany

**Keywords:** ovary arrest, fruit set, SlAGL6, tomato fruit size, CRISPR/Cas9, Solyc01g093960

## Abstract

The extreme sensitivity of the microsporogenesis process to moderately high or low temperatures is a major hindrance for tomato (*Solanum lycopersicum*) sexual reproduction and hence year‐round cropping. Consequently, breeding for parthenocarpy, namely, fertilization‐independent fruit set, is considered a valuable goal especially for maintaining sustainable agriculture in the face of global warming. A mutant capable of setting high‐quality seedless (parthenocarpic) fruit was found following a screen of EMS‐mutagenized tomato population for yielding under heat stress. Next‐generation sequencing followed by marker‐assisted mapping and CRISPR/Cas9 gene knockout confirmed that a mutation in Sl *AGAMOUS‐LIKE 6* (Sl *AGL6*) was responsible for the parthenocarpic phenotype. The mutant is capable of fruit production under heat stress conditions that severely hamper fertilization‐dependent fruit set. Different from other tomato recessive monogenic mutants for parthenocarpy, Sl*agl6* mutations impose no homeotic changes, the seedless fruits are of normal weight and shape, pollen viability is unaffected, and sexual reproduction capacity is maintained, thus making Sl*agl6* an attractive gene for facultative parthenocarpy. The characteristics of the analysed mutant combined with the gene's mode of expression imply Sl *AGL6* as a key regulator of the transition between the state of ‘ovary arrest’ imposed towards anthesis and the fertilization‐triggered fruit set.

## Introduction

Fruit development following fertilization is critical for the completion of the plant life cycle. In tomato, the ovary, which develops in concert with the rest of the flower organs (growth phase I, according to Gillaspy *et al*., 1993), ceases to undergo cell divisions shortly (1–2 days) before anthesis and enters an ‘ovary arrest’ state. Only if fertilization is successfully completed, a signal believed to be produced by the young embryo provokes the ovary to resume growth. This growth involves initially a phase of rapid cell division and expansion (designated phase II or ‘fruit set’) for 5–10 days (Bohner and Bangerth, 1988; Varga and Bruinsma, 1986), and subsequently (during phase III) growth is driven mainly by cell enlargement concomitant with nuclear polyploidization (Chevalier *et al*., 2014, and references therein). Once reaching full size, ripening processes initiate.

The default programme of fertilization‐dependent fruit development ensures that resources are not wasted sustaining purposeless fruit development, whereas parthenocarpy, that is fertilization‐independent seedless fruit development, is a counterproductive trait in all the sexually reproducing plant species. Sexual reproduction entails that the hypersensitivity of the microsporogenesis process, and the mature male gametes to moderately high or low temperatures, and to extreme humidity or light intensity (El Ahmadi and Stevens, 1979; Mesihovic *et al*., 2016; Picken, 1984; Sato *et al*., 2006), is a major hindrance for year‐round fertilization‐dependent tomato yielding. Consequently, breeding for parthenocarpy is considered a valuable goal especially in the context of maintaining sustainable agriculture in the face of global warming (Ariizumi *et al*., 2013; Gorguet *et al*., 2005; Ruan *et al*., 2012). Other advantages of parthenocarpy relate to consumers’ preference of seedless over seeded fruits, improved fruit quality due to elevated content of total soluble solids (TSS) (Carmi *et al*., 2003; Casas Diaz *et al*., 1987; Falavigna *et al*., 1978; Ficcadenti *et al*., 1999) and saving of energy invested in separating the seeds from processed products.

Since tomato and other vegetables that could benefit from parthenocarpy are commonly propagated from seeds, hence only genetic sources for facultative parthenocarpy, where seeded fruits can develop following successful fertilization (Varoquaux *et al*., 2000), are of practical value. Presently, the most extensively characterized nontransgenic sources for facultative parthenocarpy in tomato are as follows: the three monogenic sources, *pat* (Beraldi *et al*., 2004; presumably a mutated Solyc03g120910, Selleri, 2011; Soressi and Salamini, 1975), *procera* (a mutated Sl*DELLA,* Bassel *et al*., 2008) and *entire* (mutated Sl*AUX/IAA9,* Mazzucato *et al*., 2015; Saito *et al*., 2011), all of which manifest undesired pleiotropic effects; and the three digenic sources, *pat*‐2 (Hazra and Dutta, 2010; Vardy *et al*., 1989), IL5‐1 and IVT‐line 1 (Gorguet *et al*., 2008), all manifesting acceptable parthenocarpic phenotype; and the inferior oligogenic source *pat‐3/pat‐4* (Nuez *et al*., 1986; Philouze and Maisonneuve, 1978). Despite the importance of this trait, exploitation of these mutants in breeding programmes is still rather limited. Some of them are associated with mild or severely undesirable pleiotropic effects (e.g. Ariizumi *et al*., 2013; Carrera *et al*., 2012; Lin *et al*., 1984; Mazzucato *et al*., 1998; Philouze, 1989). And introgression of a digenic source is much more laborious, especially since the identity of the genes underlying any of these three sources was not reported so far. Besides their applicative importance, parthenocarpic mutants are indispensable in the study of the mechanism underlying ‘ovary arrest’ at pre‐anthesis and its fertilization‐triggered release leading to fruit set.

In the present study, we demonstrate that mutated alleles of the MADS‐box gene Sl*AGL6* enable tomato yielding under heat stress. The mutations confer facultative parthenocarpy manifested in the development of seedless fruits comparable in both weight and shape to wild‐type (WT) seeded fruits and that without pleiotropic effects, thus making it a novel valuable source for parthenocarpy in tomato. The pivotal role of Sl*AGL6* in controlling the transition from the state of ‘ovary arrest’ to fertilization‐triggered fruit set is also discussed.

## Results

### Line 2012 is a new monogenic recessive mutant for parthenocarpy

A chemically, EMS‐mutagenized M_2_ population generated in the M82 cultivar (generated by J. Hirschenhoren and Y. Kapulnik, The Volcani Center, ARO) was screened for mutants yielding under extremely high temperatures (Figure S3a), which prevented fertilization‐dependent fruit set, as described in Data S1. Family No. 2012 included two plants that set high‐quality parthenocarpic fruits with good jelly fill under these conditions, whereas the parental line set only tiny, hollow fruits, commonly dubbed ‘nuts’.

**Figure 1:**
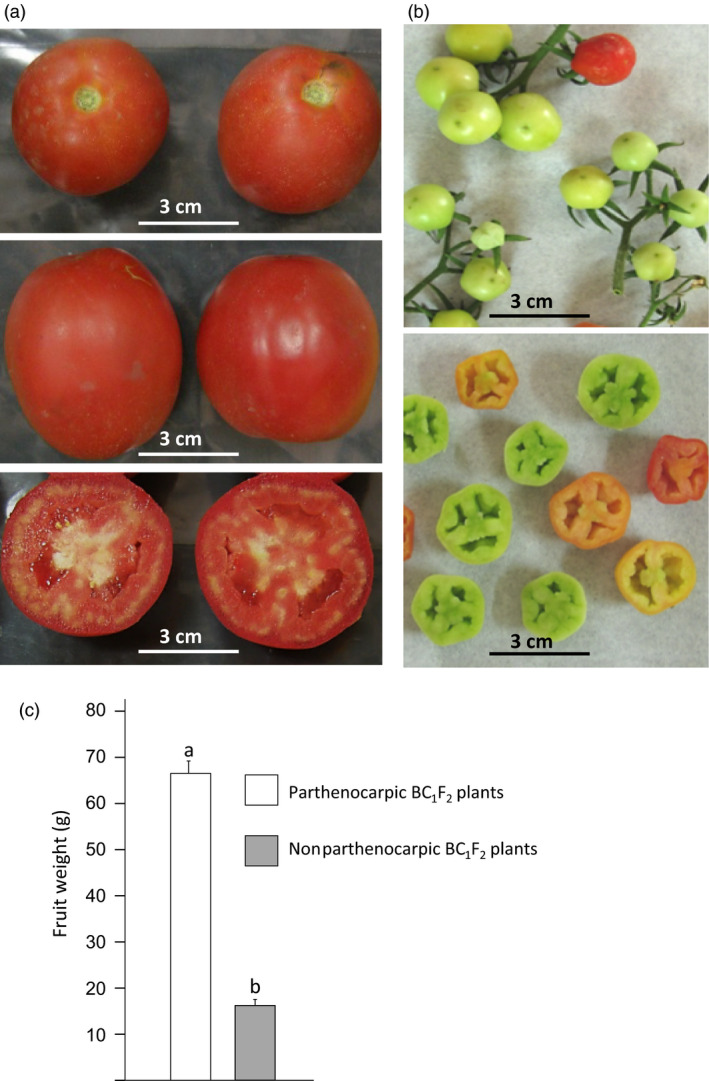
The 2012 mutation enables parthenocarpic tomato fruit development under heat stress. In BC_1_F_2_ population segregating for the 2012 mutation, when yielding in the late summer: (a) parthenocarpic siblings set high‐quality seedless fruits, whereas (b) M82 plants set small distorted fruitlets. (c) Within the segregating BC_1_F_2_ population, the weight of the fruit harvested from 23 parthenocarpic plants was significantly higher than that from 24 nonparthenocarpic siblings (*t*‐test, *P* < 0.001).

One of these two plants served to pollinate emasculated flowers of M82 plant, which set seeded fruits. These BC_1_ plants were not parthenocarpic. However, 7 out of 40 BC_1_F_2_ progenies set seedless fruits under the extremely hot conditions prevailing in the late summer (Figure S3b) when the parental line M82 managed to set tiny ‘nuts’ fruitlets only (Figure 1a *vs*. b). This indicated that the trait is governed by a single recessive mutation. Further, out of 30 plants from the same BC_1_F_2_ population grown in the winter (Figure S3c), in a nonheated glass house, nine set seedless fruits, whereas the rest of the siblings set no normal fruits, indicating that the mutation enables parthenocarpic fruit development also under temperatures too low to allow fertilization‐dependent fruit set.

### Mapping the 2012 mutation

To map the mutation, we chose to adopt the bulk segregation approach (Michelmore *et al*., 1991) and perform Illumina sequencing of two genomic libraries one coming from a bulk of 2012 BC_1_F_2_ parthenocarpic plants and the other from their nonparthenocarpic siblings. For this purpose, BC_1_F_2_ plants were grown again in the late summer when the day temperatures were high enough (Figure S3d) to seriously damage microsporogenesis and hence prevented fertilization‐dependent fruit development. Under these conditions, at the date of harvest, the parthenocarpic plants produced high‐quality red parthenocarpic fruits with good jelly fill, whereas the nonparthenocarpic (NP) siblings produced only small distorted, green puffy ‘nuts’ fruits, which weight was significantly smaller than that of the red fruits harvested from their parthenocarpic siblings (Figure 1c).

The genomic library coming from a pool of 20 BC_1_F_2_ plants characterized as ‘clearly parthenocarpic’ was designated ‘2012 library’, and the other coming from a pool of 23 of their siblings characterized as ‘clearly nonparthenocarpic’ was designated ‘NP (nonparthenocarpic) library’. Bioinformatics analysis of the sequenced libraries was performed as detailed in Data S1. This analysis pointed to a segment of 3.85 million nucleotides in chromosome 1, spanning between SL2.5ch01:85115654 and 88965277, as the likely location of the mutation (Table 1). The analysis revealed nine homozygous mutated single nucleotide polymorphisms (SNPs) in this region in the ‘2012 library’ that were heterozygous in the ‘NP library’, that is only 19–37% of the reads in this library showed the mutated alleles, while the others included the expected wild‐type (WT) allele.

**Table 1:** Description of the SNPs along the predicted location of the 2012 mutation in chromosome 1. Bioinformatics analysis of two sequenced libraries, one representing nonparthenocarpic (NP) and the other parthenocarpic (2012) siblings from the BC_1_F_2_ population, provided the presented numbers of WT and mutated nucleotide reads for each of the nine SNPs in each of the two libraries. ORF – open reading frame

SNP	SNP No.^a^	Position on Ch 1 (M82) SL2.50	No. WT reads/No. mutated reads		Number and annotation of the mutated gene (in parenthesis: position of the SNP)
			NP library	2012 library	
I	1	85 115 654	52G/28A	0G/60A	Solyc01g091480; Armadillo repeat kinesin 2 (first intron)
II	2	85 400 236	59A/29C	0A/67C	Solyc01g091860, SET domain protein, possibly involved in peptidyl‐lysine monomethylation (10th intron)
III	3	85 536 662	32G/25A	0G/53A	Solyc01g093960, Agamous‐like MADS‐box 6 (ORF premature stop)
IV		85 785 954	32G/25A	0G/53A	Solyc01g094230, Protein phosphatase‐2C (ORF, silent)
V	4	86 070 972	55T/16C	0T/71C	Intergenic
VI		86 587 877	25G/6A	0G/55A	Solyc01g095250, Chitinase, Glycoside hydrolase X2 (ORF, mis‐sense)
VII		87 367 821	22T/13A	0T/45A	Intergenic
VIII	5	88 007 968	61C/24T	0C/99T	Solyc01g097030, MUSTANG transposase Zn fingers (first intron)
IX	6	88 965 277	49A/26G	0A/65G	Intergenic
Total Distance (bp)		3 849 623			

a The corresponding no. of the SNP when genotyped by DYN R&D.

To restrict the location of the mutation underlying the 2012 mutant, we tested, in two segregating populations, for cosegregation of SNPs dispersed along the chromosomal interval suggested as the mutation location (Table 1, column 2), with the parthenocarpic phenotype. Cosegregation with six SNPs was examined in a testcross (TC) population (designed to segregate in a 1 : 1 ratio of parthenocarpic and nonparthenocarpic plants) which was allowed to set fruit under heat stress (Figure S3e). As shown in Table 2a, this analysis eliminated the candidacy of the mutations represented by SNP Nos. 1, 5 and 6 as they are not closely linked with parthenocarpy. To further zoom in on the location of the mutation, a 2012 BC_2_F_2_ population was similarly analysed for cosegregation of the parthenocarpic phenotype with the mutated version of SNP Nos. 2 and 3. This analysis, which is summarized in Table 2b, eliminated the candidacy of SNP No. 2, since six nonparthenocarpic plants were homozygous for its mutated version. Because of the incomplete linkage between the parthenocarpic phenotype and the mutated SNP No. 3, plants homozygous for mutated SNP No. 3 were genotyped also for SNP No. 4. Yet SNP No. 4 remained a less likely candidate because the three nonparthenocarpic plants were homozygous also for its mutated version (Table 2b). Furthermore, the progenies (BC_2_F_3_) of these three plants manifested clear parthenocarpy when allowed to set fruit in the winter, under suboptimal temperatures (Figure S3g), suggesting that the nonparthenocarpic phenotype of their parents reflects the facultative nature of the mutation.

**Table 2:** Analysis of cosegregation of the 2012 parthenocarpic mutation with candidate SNPs. (a) The testcross (TC) population was genotyped for the six SNPs specified in Table 1 (column 2), in the few cases where the genotyping was inconclusive less than 96 results are presented. (b) The BC_2_F_2_ population was genotyped for SNP Nos. 2 and 3. The 126 plants homozygous for mutated version of SNP No. 3 were also genotyped for SNP No. 4. The three nonparthenocarpic plants homozygous for mutated SNP No. 3 are also homozygous for mutated SNP No. 4. The analysed populations are detailed in Data S1. m: mutated and +: WT versions of a SNP. N.T. – not tested

SNP Site #	Distance between consecutive SNPs	Phenotype	No. of plants carrying the various SNP genotypes in:					
			(a) The TC population (*n* = 96)			(b) The BC_2_F_2_ population (*n* = 498)		
			m/m	+/m	+/+	m/m	+/m	+/+
1	0	Parthenocarpic	0	0	50	N.T	N.T	N.T
		Nonparthenocarpic.	0	0	46	N.T	N.T	N.T
2	284 582	Parthenocarpic	44	4	0	123	1	0
		Nonparthenocarpic	1	44	0	6	254	114
3	136 426	Parthenocarpic.	47	3	0	124	0	0
		Nonparthenocarpic	1	45	0	3	257	114
4	534 310	Parthenocarpic	46	3	0	120	3	0
		Nonparthenocarpic.	3	42	0	3	0	0
5	1 936 996	Parthenocarpic	44	5	0	N.T	N.T	N.T
		Nonparthenocarpic	5	41	0	N.T	N.T	N.T
6	957 309	Parthenocarpic	29	17	3	N.T	N.T	N.T
		Nonparthenocarpic	3	24	18	N.T	N.T	N.T
Total Distance (bp)			3 849 623			670 736		

Together, these analyses strongly suggested the mutated Sl*AGL6* (represented by SNP No. 3, see Table 1), as the gene underlying the parthenocarpic mutation 2012. *SlAGL6* encodes for a MADS‐box protein belonging to the type II lineage MIKC^C^, subfamily AGL6, of the MADS‐box transcription factor family (Smaczniak *et al*., 2012).

### CRISPR/Cas9‐mutated *SlAGL6* confers parthenocarpy

**Figure 2:**
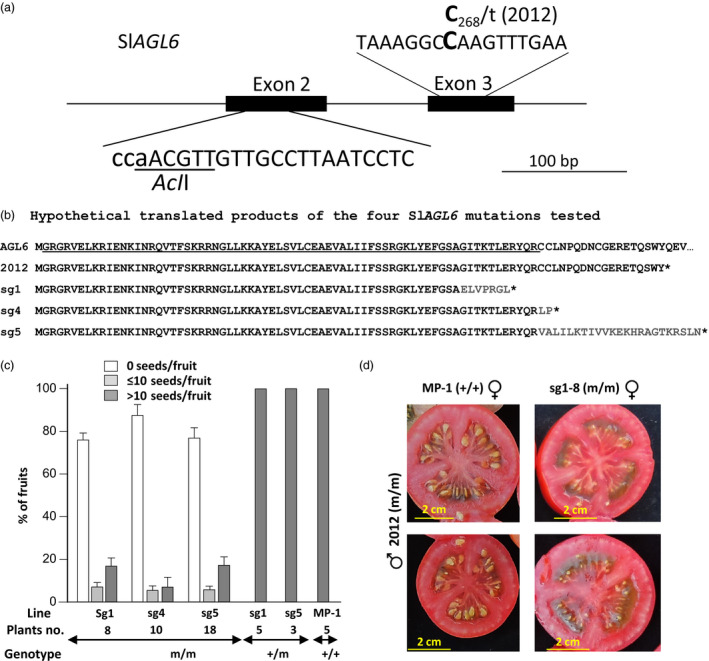
The CRISPR/Cas9‐generated Sl *AGL6* mutations are parthenocarpic. (a) The chosen target guiding sequence for CRISPR/Cas9 modification in exon 2 of SlAGL6 is presented, the PAM (its reverse complement) is depicted in lower case letters, and the AclI restriction site expected to be destroyed by Cas9‐induced mutations is underlined. The EMS‐induced mutation 2012 in exon 3 is also presented. (b) The hypothetical translated products of the four SlAGL6 mutations tested: the EMS‐induced 2012 and the CRISPR/Cas9‐generated mutations sg1, sg4 and sg5. For the WT SlAGL6 protein, only the first 92 amino acids (AA) are presented, and the MADS‐box domain is underlined. Grey coloured AAs differ from those of the WT protein. The asterisk (*) denotes premature stop codon. (c) The CRISPR/Cas9‐derived Sl *AGL6* mutations sg1, sg4 and sg5 all lead to facultative parthenocarpy. Presented is the mean rate (%) ± SEM of seedless, underseeded and seeded fruits in plants homozygous, or heterozygous for the mutated allele, and in the parental line MP‐1. (No heterozygous sg4 progenies were found among the screened ones). d) F_1_ hybrid between plant homozygous for the 2012 mutated allele of Sl *AGL6* and plant homozygous for the sg1 mutated allele produced seedless fruits, whereas a hybrid between the same 2012 plant and MP‐1 produced seeded fruits only, testifying to allelism of 2012 and sg1 mutated version of Sl *AGL6*.

To confirm that the mutated *SlAGL6* is the gene underlying the 2012 parthenocarpy, CRISPR/Cas9 technology was exploited to knockout the *SlAGL6* gene (*Solyc01g093960*). Synthetic gRNA was designed to target the second exon of *SlAGL6* (Figure 2a). It was incorporated into a Cas9 expressing binary vector and transformed into tomato line MP‐1.

Progenies of three *R*_0_ plants designated sg1, sg4 and sg5 differing in the nature of their mutation (Data S1), but all leading to premature stop codon (Figure 2b), were chosen for analysis of their ability to set parthenocarpic fruits. *R*_1_ plants genotyped as heterozygous (+/m) or homozygous (m/m) for mutated *SlAGL6* were grown in a greenhouse, side by side with the parental line MP‐1. Red fruits were picked, weighed and analysed for seeds bearing. As demonstrated in Figure 2c, heterozygous progenies produced seeded fruits only, whereas the progenies homozygous or bi‐allelic for mutated versions of Sl*AGL6* set mostly parthenocarpic fruits, as well as a few underseeded fruits (bearing up to 10 seeds), and fruits containing more than 10 seeds (defined as seeded fruits). Thus similar to the 2012 mutant, they are manifesting facultative parthenocarpy. Besides being seedless, the parthenocarpic fruits are similar in shape and jelly fill to those of the parental line fruits or their heterozygous seeded siblings (Figure S1).

The conclusive proof for allelism of the 2012 mutation and the CRISPR/Cas9‐generated Sl*AGL6* mutants came from the parthenocarpic phenotype of most of the fruits produced on F_1_ hybrid between sg1 plant homozygous for mutated Sl*AGL6* and 2012 plant homozygous for mutated SNP No. 3, whereas F_1_ hybrid between MP‐1 (+/+) and the same 2012 plant produced seeded fruits only (Figure 2d).

**Figure 3:**
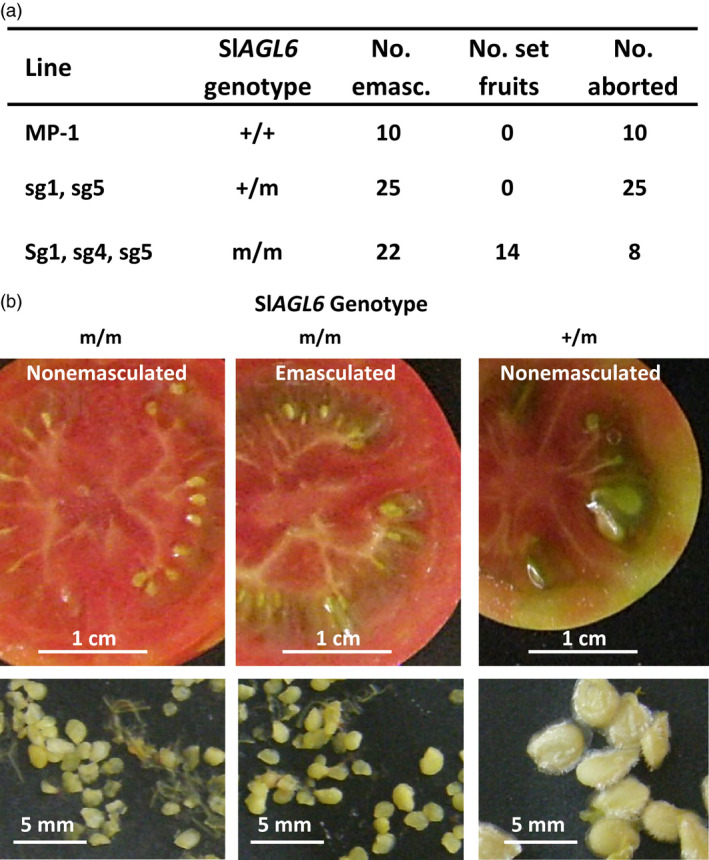
The CRISPR/Cas9‐derived Sl *AGL6* mutations induce vegetative parthenocarpy. (a) Fruit set from flowers emasculated at pre‐anthesis from plants of the indicated Sl *AGL6* genotype. (b) The enlarged ovules collected from red fruits developed from nonemasculated or emasculated flowers developed on a plant homozygous for CRISPR/Cas9‐mutated Sl *AGL* 6 allele are comparable in size and appearance. Both are similarly smaller than true seeds collected from fruits of a heterozygous (+/m) sibling. The three presented fruits were harvested on the same date.

If the observed parthenocarpy is true (‘vegetative’), namely requires no external trigger, it will set fruit under pollination restrictive conditions; if the mutant is ‘stenospermocarpic’, that is requires an external stimulus (provided, e.g., by damaged pollen or the young embryos before their abortion), it implies that this parthenocarpy still relies on pollination, even though an unsuccessful one (Varoquaux *et al*., 2000). To distinguish between the two, flowers were emasculated at pre‐anthesis and tested for fruit set. As shown in Figure 3a, all the emasculated flowers of the *R*_1_ heterozygous progenies and MP‐1 aborted, whereas over 60% of the emasculated flowers of mutated homozygous progenies set fruits, testifying to the true vegetative nature of the Sl*agl6*‐induced parthenocarpy. In agreement, the enlarged ovules collected from fruits developed from emasculated flowers are similar in size and appearance to those collected from nonemasculated parthenocarpic fruits, both of which are substantially smaller than normal seeds (Figure 3b).

### Mutated Sl*AGL6* maintains fruit weight

In many cases, parthenocarpy was claimed to reduce fruit size compared to that of seeded fruits. As a first step towards assessing a possible penalty of the *SlAGL6* mutation on yielding parameters, its effect on fruit weight was examined in three different populations/genetic backgrounds: (i) the segregating 2012 BC_2_F_2_ population, in the background of the determinate cultivar M82, (ii) the segregating F_2_ population derived from a cross between the big fruit semi‐determinate cultivar Marmande and parthenocarpic 2012 plant, and (iii) *R*_1_ progenies of the CRISPR/Cas9‐derived line sg1 in the indeterminate MP‐1 line background.

**Figure 4:**
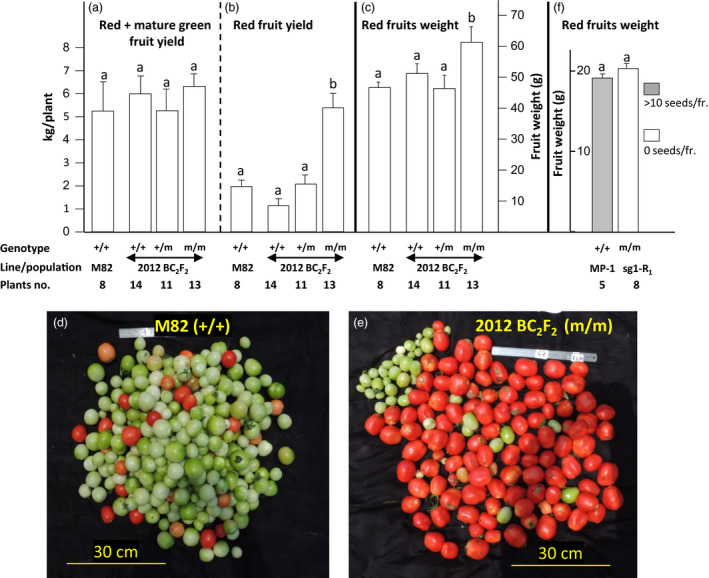
Effect of Sl *AGL6* genotype on yield components in the M82 and MP‐1 backgrounds, under ambient temperatures. (a–c) Analysis of 2012 BC_2_F_2_ plants with different genotypes of SNP No. 3 (Sl *AGL6*) compared to line M82. (a) Yield potential, defined as the yield (kg/plant) of all fruits reaching at least the mature green stage, is not affected by the mutation (one‐way analysis of variance, *P* = 0.75); (b) compared to M82, only siblings homozygous for the mutation produce significantly (*t*‐test, *P* < 0.001) higher yield of marketable red fruit; (c) the weight of their red fruits is significantly (*t*‐test, *P* < 0.05) higher than that of M82 At the date of harvest; (d) in M82, most of the harvested fruits were still green, while (e) in (m/m), plant most of them were red ripe; and (f) parthenocarpic fruits of line sg1 plants homozygous for the mutation (at *R*_1_ generation) are comparable in weight to seeded MP‐1 fruits (*t*‐test, *P* = 0.946). In panels a, b, c and f, columns accompanied by different lowercase letters differ significantly.

The effect of the mutation on fruit weight and yielding potential was assessed on the same 2012 BC_2_F_2_ plants used to map the mutation (Table 2). This population grew under near‐optimal temperatures for fertilization‐dependent fruit setting (detailed in Data S1 and Figure S3f). Three and a half months after planting in the net house, all the red, breaker and mature green fruits were harvested from 11 to 14 plants carrying the following three alternative genotypes of Sl*AGL6*: homozygous WT (+/+), heterozygous (+/m) or homozygous mutated (m/m) allele, as well as from eight M82 plants. As shown in Figure 4a, the yielding potential, expressed as the yield including mature green, breaker and red fruits, harvested from the plants homozygous for the mutated allele does not differ from that of line M82, or the siblings either homozygous (+/+) or heterozygous (+/m) for the WT Sl*AGL6* allele. Further, the plants homozygous for the mutated allele were characterized by a profoundly earlier and more concentrated yielding, manifested in a significantly higher yield of red fruits at the date of harvest (Figure 4b, and d *vs*. e). The average weight of the red fruits developed on the (m/m) plants is significantly higher than that of the seeded red fruits harvested from the parental line M82, or its (+/+) and (+/m) siblings (Figure 4c).

In order to start estimating the potential of the 2012 mutation to support development of parthenocarpic fruits also when introduced into large fruit background, 2012 plant was crossed with the medium–large, multilocular fruit, semi‐determinate open variety Marmande (www.rareseeds.com/marmande-tomato/), and the effect of the mutation on parthenocarpic fruit weight was estimated in the segregating F_2_ population (described in Data S1). Fruit weight is governed by several QTLs (Tanksley, 2004), and in segregating F_2_ population, the average fruit weight is always similar to that of the small fruit parent, with only small portion of the progenies close in weight to that of the big fruit parent (Lippman and Tanksley, 2001; Perry, 1915). Thus, we examined whether, in this F_2_ population, the mutation affects fruit weight and the tendency to generate parthenocarpic fruits close in size to the big fruit parent. Analysis performed on a small population including 48 F_2_ plants grown in 5‐L pots in a greenhouse indicated that among the 11 progenies homozygous for the mutated Sl*AGL6* (SNP No. 3), two produced seedless fruits of weight higher than that of the seeded fruits of the big fruit parent, and the average fruit weight of the mutated progenies was somewhat higher than in their nonmutated siblings (Figure S2a). Plants from the same F_2_ population were also grown in the soil, in a net house, next to the BC_2_F_2_ population described above. Fruits were picked and analysed from the 26 homozygous mutated plants. As shown in Figure S2b, c, two out the five plants with the highest average fruit weight showed a very strong parthenocarpic phenotype, as nearly all of their fruits were seedless. These analyses indicate that the mutation does not prevent the parthenocarpic F_2_ progenies from reaching weight similar to that of the big fruit parent.

Surprisingly, 12 out of the 13 tested *R*_1_ progenies of plant sg1 were found to be devoid of the transgenic cassette, while all the tested progenies of plants sg4 and sg5 contained it. Absence of the transgene enabled to assess in sg1 *R*_1_ progenies the effect of the Sl*AGL6* mutation *per se,* on fruit weight. As shown in Figure 4f, the weight of seedless fruits harvested from sg1 *R*_1_ progenies homozygous for the mutation was comparable to that of seeded fruits developed on MP‐1 plants grown side by side under the same ambient conditions.

### Sl*agl6* improves yielding under heat stress

**Figure 5:**
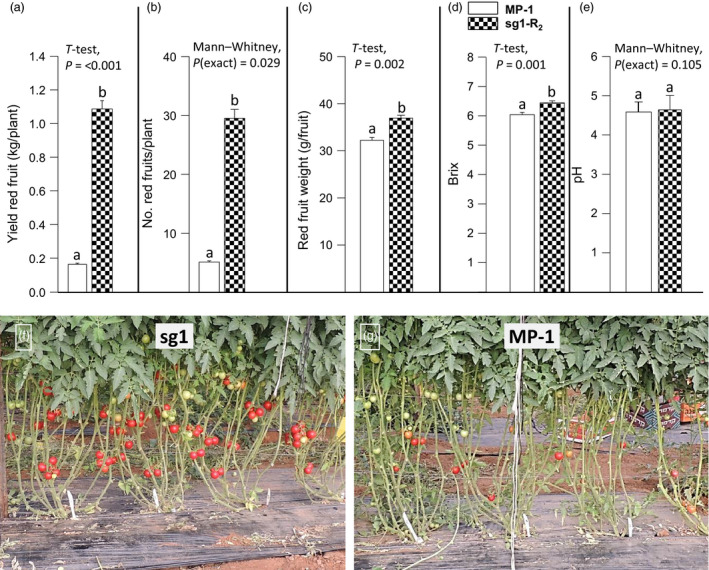
Sl*agl6* parthenocarpy improves yield under heat stress. Compared to line MP‐1, line sg1 manifests significantly higher: (a) red fruit yield, (b) number of red fruits, (c) red fruit weight and (d) Brix, while (e) the fruit pH remains unchanged. Data presented in a‐e are derived from experiment performed on four replicates, as detailed in Data S1. (f, g) Difference in fruit load between MP‐1 (f) and sg1 (g) plants partly defoliated and photographed before harvest. Growth and climatic conditions are detailed in the results section. Line sg1 (at *R*_2_) is homozygous for the sg1 mutation depicted in Figure 2b and devoid of the *Cas9* cassette.

Yielding under natural heat stress conditions was examined comparing MP‐1 and sg1 line homozygous for Sl*agl6* and devoid of the Cas9 cassette (at R_2_). Plants were planted in a net house on 20 April 2016 and the first harvest was performed 67 days later (as detailed in Data S1). During the months of May and June, the day temperatures were very high including a 3 days long spell (between 14 and 16 of May 2016) of extremely high temperatures (maximum day temperature 38 °C and above, Figure S3h). These naturally occurring heat stress conditions fall under the definition of ‘chronic mild heat stress’ known to hamper microsporogenesis and hence fertilization‐dependent fruit set (see Mesihovic *et al*., 2016; and references therein). As demonstrated in Figure 5a, under these climatic conditions, the red fruit yield of the parental line MP‐1 was significantly lower than that of line sg1 (ca. 85% lower). This difference reflects mainly a dramatic difference in the number of fruits produced (Figure 5b, f, g), which was 83% lower in line MP‐1, and also a significant lower fruit weight in the latter (Figure 5c), although by 13% only. Similar to other parthenocarpic mutants (e.g. Carmi *et al*., 2003; Casas Diaz *et al*., 1987), the TSS content, expressed as Brix, of red ripe seedless fruits was significantly higher than that of seeded fruits of MP‐1 (Figure 5d), while the acidity (pH) of the fruits remained similar (Figure 5e).

### Sl*AGL6* mode of expression

**Figure 6:**
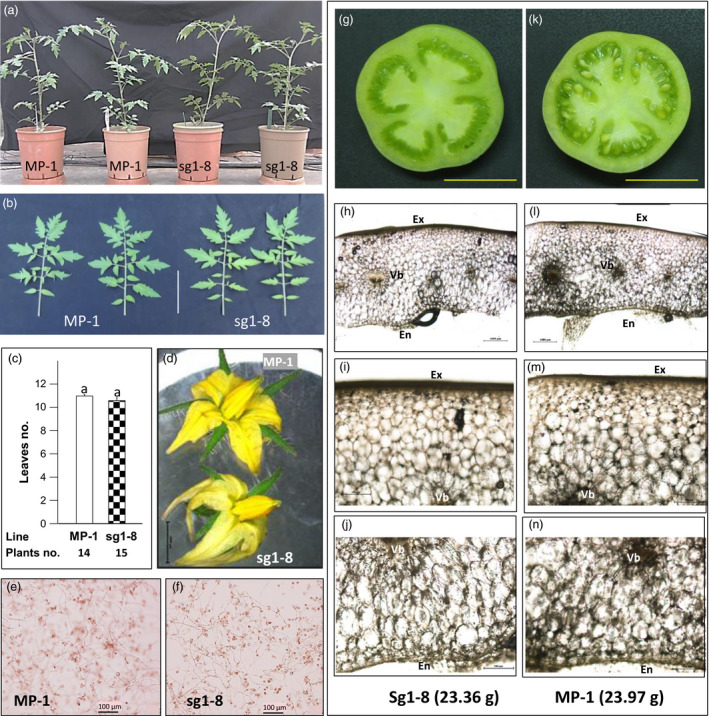
Phenotypic similarity between MP‐1 and Sl *AGL6*‐mutated line sg1‐8. (a) The plants do not differ in growth habit; (b) the shape of the leaves is similar, scale = 8 cm; and (c) the first inflorescence appeared after a similar number of true leaves (Mann–Whitney rank sum test, *P* = 0.371). (d) the flowers are similar except the petals of sg1‐8 being paler and somewhat narrower and longer than those of MP‐1, scale = 5 mm. (e, f) Pollen fertility is similar. Presented are *in vitro* germinated pollen grains with similarly elongated pollen tubes, photographed after 18‐h incubation, scale = 100 μm. (g–n) The pericarp of parthenocarpic fruit of line sg1‐8 (g‐j) is similar to that of seeded fruit of MP‐1 (k‐n) of similar weight (ca. 23.6 g) and size (g, k scale = 2 cm). (h, l) The pericarp is of similar width and shape, scale = 1000 μm. The cells in the layers between the vascular bundle rim and the exodermis (the exocarp) (i, m) and those between the vascular bundle rim and the endodermis (endocarp) (j, n) are of similar appearance. In i, j, m, n, scale = 500 μm. Photographs (h–j) and (l‐n) are of thin freehand transverse sections taken from the middle (equator) of the nearly mature green fruits presented in g and k, and photographed under light microscope. Line sg1‐8 is described in legend to Figure 5. Ex, exodermis; En, endodermis; Vb, vascular bundle.

To gain additional insight into the function of Sl*AGL6*, its expression was queried in publically available data and complemented by quantitative RT‐PCR of developing ovaries. According to the Expression Atlas of Tomato Tissues (http://tomatolab.cshl.edu/~lippmanlab2/allexp_query.html, Park *et al*., 2012; Tomato Genome Consortium 2012), Sl*AGL6* is highly expressed only in the flower meristem, flower bud and open flower, yet it sharply declines in the developing fruit (Figure S4a). This explains why the Sl*agl6* mutation does not affect vegetative development, transition to reproductive stage, pollen viability, or fruit size and shape (Figure 6a–c, e–n). However, despite the reported expression in the flower meristem, the flower bud and the flower (Figure S4a), the only subtle difference noticed in the flowers is that the petals are paler and somewhat narrower and longer than in the WT (Figure 6d).

In agreement with the Expression Atlas of Tomato Tissues, four transcriptomic analyses concerning tomato fruit set reported that relative to pre‐anthesis (−2 days postanthesis, DPA) (Tang *et al*., 2015; Wang *et al*., 2009) or anthesis (Pattison *et al*., 2015; Zhang *et al*., 2016), Sl*AGL6* expression sharply declines in fertilized fruit at 4‐5DPA (Figure S4b,c,d). However, in unpollinated emasculated ovaries, it scarcely declined (by 1.4‐fold only, Figure S4b), suggesting that its decline following fertilization is inherent to fruit set.

**Figure 7:**
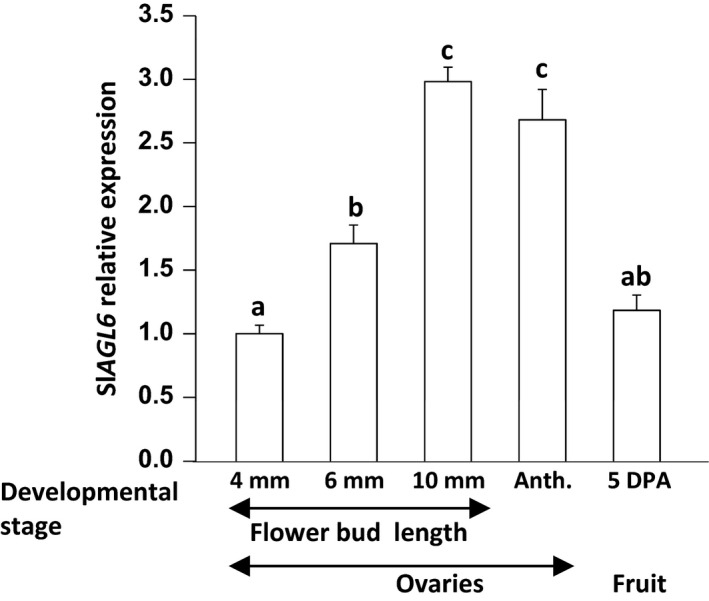
Relative expression of Sl *AGL6* during ovary development in M82 cultivar. qRT‐PCR analysis of *SlAGL6* in developing ovaries (developmental stage defined by flower bud length), ovaries at anthesis (Anth.) and young fruits harvested 5DPA. Relative expression levels were normalized to *SlTIP41* (Solyc10g049850) as the reference gene and calculated by the comparative delta delta Ct (ΔΔCt) method. The analysis was performed on three biological replicates. Columns accompanies by different lowercase letters differ significantly (Tukey–Kramer HSD test, *P* ≤ 0.01).

To examine Sl*AGL6* mode of expression during growth phase I, its expression was quantified in developing ovaries of line M82. As demonstrated in Figure 7, Sl*AGL6* expression elevates in the developing ovaries, and it peaks towards the stage of ‘ovary arrest’ (10‐mm‐long buds correspond to pre‐anthesis) and remains high at anthesis. Yet 5DPA it sharply declines to the level found in ovaries of the young 4‐mm‐long flower buds, hence associating ‘ovary arrest’ with elevated Sl*AGL6* expression and fruit set with its decline. Genetic variation between M82 and Micro Tom or *Solanum pimpinellifolium* might explain the higher fold decline between anthesis and 4DPA reported before (Pattison *et al*., 2015; Tang *et al*., 2015; Wang *et al*., 2009), whereas smaller variation between M82 and Moneymaker could account for the observed similarity in fold decline (Figure 7 *vs*. Figure S4d, queried from Zhang *et al*., 2016).

Evidence for preferential sublocalization of the Sl*AGL6* transcript within the ovules of the mature arrested ovary was provided by Pattison *et al*. (2015). Following transcriptomic analysis of laser‐captured tissues, they show that Sl*AGL6* level in the ovules is at least fourfold higher than in the other tissues comprising the ovary. However, at 4DPA, its expression in the embryo is already 15‐fold lower than in the ovule (Fig. S4c). This finding was further corroborated by Zhang *et al*. (2016) (Fig. S4d).

## Discussion

### Loss of Sl*AGL6* function results in facultative parthenocarpy that ensures fruit production under high temperatures

After isolating the EMS‐induced 2012 parthenocarpic mutant (Figure 1a *vs*. b), next‐generation sequencing (Table 1) followed by marker‐assisted mapping (Table 2) and CRISPR/Cas9 gene knockout (Figure 2) confirmed that mutated Sl*AGAMOUS‐LIKE 6* confers facultative parthenocarpy in tomato.

To fully determine whether mutated Sl*AGL6* imposes any unacceptable penalty on yielding potential or fruit characteristics, it waits to be introduced as a single mutation into elite tomato cultivars and tested when grown under ambient environmental conditions and established horticultural practices. Nonetheless, in three different genetic backgrounds, the weight of Sl*agl6* seedless fruits was comparable or even higher than that of the seeded WT ones (Figures 4c, f, 5c, Figures S1 and S2), and yielding potential was comparable to that of the parental cultivar M82 (Figure 4a).

The exact conditions favouring seed setting in the mutated plants under fertilization permissive conditions remain to be elucidated. Vigorous inflorescence vibration resulted in many seeded fruits from plants that otherwise set mainly seedless ones (data not shown). In the absence of intentional vibration, enhanced tendency was clearly associated with two parameters: first, the small fruits developed on old plants, frequently bear seeds. This is an unusual phenomenon, as it was shown that seeded tomato fruits are larger than underseeded ones (Carmi *et al*., 2003; Imanshi and Hiura, 1975; Varga and Bruinsma, 1976). Second, fruits that set at temperatures mildly lower than optimal were frequently found to contain seeds. In both cases, seed production presumably reflects conditions slowing the rate of ovary expansion into fruit, thus allowing the pollen grains to complete germination, elongation and fertilization of ovules before the style is detached from the otherwise rapidly expanding ovary/fruit. The genetic background apparently affects the facultative manifestation as well: while in 50% (13/26) of the Slagl6/Slagl6 F_2_ progenies of the 2012 × Marmande hybrid, grown under ambient conditions, 8/8 fruits tested per plants were seeded (Figure S2b), only 2.4% (3/126) of the Slagl6/Slagl6 plants of the 2012 BC_2_F_2_ population grown side by side were completely seeded (see Table 2b). Although not tested during the relevant flowering period, genetic differences in anther dehiscence under the given humidity conditions, the rate of ovary enlargement pre‐ and postanthesis and/or the duration of stigma receptivity could be among the factors underlying the observed difference between these genetic backgrounds.

The ability of the Sl*agl6‐*induced parthenocarpy to solve the problem of yielding under fertilization restrictive conditions such as imposed by chronic mild heat stress (Mesihovic *et al*., 2016) is its most important agronomic attribute. When challenged by continuous mild heat stress, which was worsen by a 3‐day spell of acute stress (Figure S3f), the mutated line yielded over sixfold higher than the parental line (Figure 5a) and that mainly because of the profoundly higher number of flowers that set (seedless) fruits under these fertilization restrictive conditions (Figure 5b, f, g). The demonstrated capability of Sl*agl6* to yield under microsporogenesis restrictive conditions (Sato *et al*., 2006), together with the ripening uniformity along consecutive trusses exhibited in the M82 determinate background (Figure 4b, d‐e), makes Sl*agl6* particularly suitable for breeding of processing tomato cultivars. This is because under fluctuating climatic conditions, it maximizes the marketable yield that can be obtained in a single mechanical harvest.

### Sl*agl6* is an attractive gene for parthenocarpy

Three digenic sources for parthenocarpy which manifest only mild or no adverse pleiotropic effects and hence of practical value are as follows: *pat‐2* (Hazra and Dutta, 2010; Vardy *et al*., 1989), IL5‐1 and IVT‐line 1 (Gorguet *et al*., 2008) (as reviewed by Ariizumi *et al*., 2013). A reliable comparative assessment of the horticultural/parthenocarpic performance of these three sources with that of the Sl*agl6* mutation waits the identification of the mutated genes underlying these sources and the introducing of all of them to a common genetic background. Nonetheless, different from them, Sl*agl6* is a single recessive source for facultative parthenocarpy, which is not allelic to any of these three, since none of them was suggested to map to chromosome 1 (Gorguet *et al*., 2008; Nunome *et al*., 2014). Taken together, the simple mode of inheritance, the lack of pleiotropic effects (Figure 6) or adverse effects on fruit weight or shape (Figures 4c, f, 5b, 6g‐n, and Figures S1 and S2), the true vegetative nature of the induced parthenocarpy (Figure 3) and its facultative manifestation make *Slagl6* an attractive single recessive gene for parthenocarpy. The CRISPR/Cas9 technology now enables expeditious integration of Sl*agl6* into any elite cultivar of interest. That can be done either by direct CRISPR/Cas9‐mediated Sl*AGL6* knockout in the two parents of the elite hybrid cultivars, if they are amenable for transformation, or introgressed by backcrossing.

### Sl*AGL6* functions before and after fertilization

Comprehensive transcriptomic studies of tomato fruit set found significant changes in the expression of hundreds of genes, including several down‐regulated MADS‐box genes (e.g. Pattison *et al*., 2015; Tang *et al*., 2015; Wang *et al*., 2009; Zhang *et al*., 2016). Among them, Sl*AG1* and Sl*AGL6* were suggested to play an important role in fruit set (Wang *et al*., 2009), and so was also the *tomato MADS‐box 29* (*TM29*) (Ruan *et al*., 2012). However, experimentally supported identification of any transcription factor actually involved in the regulation of ‘ovary arrest’ unless it is fertilized is still missing (Ruan *et al*., 2012). The severe homeotic malformations of the seedless fruit developed following silencing *TM29* (Ampomah‐Dwamena *et al*., 2002) or Sl*AG1* (Gimenez *et al*., 2016; Pan *et al*., 2010; Pnueli *et al*., 1994) indicate a role in stamen and carpel development rather than as activators of the normal fertilization‐triggered fruit set, and experimental support for the Sl*AGL6* suggested function was not provided.

**Figure 8:**
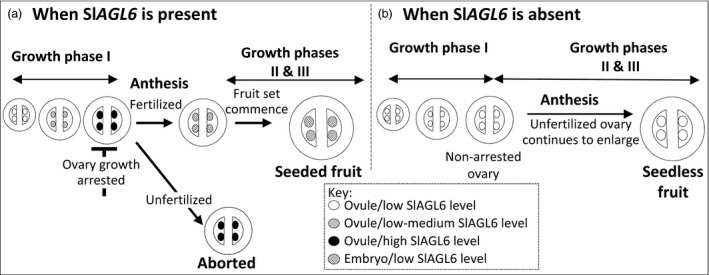
A model depicting the key role of SlAGL6 in the regulation of fertilization‐dependent fruit set. (a) Low level of Sl *AGL6* in the ovules of the young ovary allows their growth until the high level, reached towards the end of growth phase I, inhibits further development of the ovary, resulting in ‘ovary arrest’. Only the fertilization‐induced down‐regulation of Sl *AGL6* in the young embryos enables the resumption of growth and production of a fully developed seeded fruit. Alternatively, (b) loss of the ‘ovary arrest’ function in the Sl*agl6* mutant allows fertilization‐independent seedless fruit development.

The studied Sl*AGL6* mutants together with relevant transcriptomic analyses identify SlAGL6 as a central player in the mechanism underpinning fertilization‐dependent fruit development. The presented data strongly indicate that the principle, if not the sole, role of SlAGL6 is to serve as a key regulator of the transition between the ‘ovary arrest’ state reached at the end of growth phase I and the fertilization‐triggered resumption of growth, that is fruit set. The information gathered in the current study led us to propose a model (Figure 8) according to which Sl*AGL6* accumulation at pre‐anthesis, most likely in the maturing ovules, plays a pivotal role in the induction or at least retention of ‘ovary arrest’. At anthesis, successful ovule fertilization signals for Sl*AGL6* down‐regulation, and once its suppressive effect is alleviated, the ovary/fruit growth is resumed and continues to its full growth potential.

The reasoning behind the proposed model is as follows: first, although according to the Expression Atlas of Tomato Tissues (http://tomatolab.cshl.edu/~lippmanlab2/allexp_query.html; Park *et al*., 2012; Tomato Genome Consortium 2012), Sl*AGL6* is highly expressed in the flower meristem, the flower bud and the flower (Figure S4a); besides the subtle change in the petal hue and shape, the Sl*agl6* flowers are normal, and both male and female fertile (Figure 6d, e‐f). This indicates that different from some other species where an E‐function was attributed to the AGL6 clade (Dreni and Zhang, 2016), SlAGL6 is dispensable in determining the identity of any of the flower whorls according to the extended ABC(DE) and the quartet models (Coen and Meyerowitz, 1991; Theissen, 2001; Theissen and Saedler, 2001). Second, quantification of Sl*AGL6* transcripts in developing ovaries of line M82 demonstrates that it is not highly expressed throughout growth phase I (Figure 7), but rather gradually elevates and peaks at the stage when the ovary growth is arrested. However, 5DPA it already declines to the low level found in ovaries of the young 4‐mm‐long flower buds. Although our analysis was performed on whole ovaries, based on the demonstrated preferential expression of Sl*AGL6* in the ovules at anthesis (Figure S4c, d, queried from Pattison *et al*., 2015; Zhang *et al*., 2016), it is reasonable to assume that the observed peak at pre‐anthesis represents mainly increased expression in the ovules of ovaries which growth is arrested. The lower level of the transcript in the younger growing ovaries, together with the Sl*agl6* parthenocarpy, strongly suggests that Sl*AGL6* accumulation in the mature ovules acts as a key suppressor of ovary growth beyond anthesis unless it is fertilized, thus preventing accidental development of unfertilized ovary into purposeless fruit. Lastly, the normal size and shape of the seedless fruits indicate that development of the fruit to its full growth potential relies predominantly on the removal of the SlAGL6 suppressive signal (either following fertilization or by mutation), rather than on promoting signals emitted by the developing embryos independent of Sl*AGL6* down‐regulation. Continuously emitted SlAGL6 suppressive signal from unfertilized ovules within the developing WT fruit (as suggested by Tang *et al*., 2015; see Figure S4b) could explain the often observed restricted development of underfertilized fruits (Carmi *et al*., 2003; Imanshi and Hiura, 1975; Varga and Bruinsma, 1976). This assumption is further supported by the significantly higher weight of the Sl*agl6* parthenocarpic fruits in the segregating 2012 BC_2_F_2_ population compared to that of the seeded fruits of the parental line M82 (Figure 4c), as well as that of the seedless sg1 fruits compared to the MP‐1 seeded ones when developed under heat stress (Figures 5c). In both cases, not all of the ovules are necessarily fertilized in the seeded WT fruits.

### Neofunctionalization of AGL6 in tomato

It is noteworthy that in apple (*Malus domestica*), where the fleshy fruit is a pome derived from the floral tube fused to the carpels, parthenocarpy was found to be governed by mutated Md*PI*, the homolog of Arabidopsis *PISTILLATA* (Yao *et al*., 2001), which belongs to the DEF/GLO rather than the AGL6 clade of MADS‐box genes (Smaczniak *et al*., 2012). Alternatively, parthenocarpy was not reported for any of the mutated Sl*AGL6* homologs characterized so far. In rice (*Oryza sativa*), the homeotic changes manifested by mutated Os*MADS6* testify to its role in determining floral organ and meristem identities (e.g. Duan *et al*., 2012; Li *et al*., 2011; Ohmori *et al*., 2009; Zhang *et al*., 2010). Knocking down its *Nigella damascene* homolog affected structure of sepals and petals indicating an A‐function (Wang *et al*., 2015). The *Arabidopsis thaliana* homolog (At2g45650) affects flowering time and axillary bud formation (Huang *et al*., 2012; Koo *et al*., 2010; Yoo *et al*., 2011). Interestingly, similar to Sl*agl6*, the mutated *Petunia hybrida* homolog, Ph*agl6*, caused only subtle effect on petals colour and indentation. Yet, although similar to tomato and *Arabidopsis* (Schauer *et al*., 2009), Ph*AGL6* is highly expressed in the mature ovules, in this dry capsule fruit species, as in *Arabidopsis*, parthenocarpy was not reported (Rijpkema *et al*., 2009). Thus similar to other MADS‐box genes that underwent neofunctionalization (Dreni and Kater, 2014; Scutt *et al*., 2006; Smaczniak *et al*., 2012; Zahn *et al*., 2006), in tomato, and presumably in other fleshy Solanaceae fruits, AGL6 acquired a new function, acting as the suppressor of ovary development beyond anthesis. Further identification of the signals and regulators involved in Sl*AGL6* down‐regulation and its immediate targets could point to additional candidate genes for parthenocarpy.

## Experimental procedures

### Populations for mapping the 2012 mutant

Several populations were analysed, including 2012 BC_1_F_2_, a testcross (TC) population, 2012 BC_2_F_2_ and F_2_ population derived from 2012 × Marmande cross. Generation of these populations, their growth conditions and parameters analysed in each of them are detailed in Data S1 and in Figure S3.

### SNP genotyping of 2012 derived progenies

Genotyping was performed as a service by DYN R&D Ltd (Migdal Haemek, Israel), following the melting curve SNP method (Ye *et al*., 2002). Plants were genotyped in duplicate, on two separately sampled leaves.

### Genomic DNA libraries, sequencing and bioinformatics analysis

From each of 20 plants derived from 2012 BC_1_F_2_ population, defined as ‘strong parthenocarpic’, one young leaf (ca. 150 mg FW) was picked, and from the pooled leaves, DNA was extract. Similarly, DNA was extracted from a pool of leaves sampled at the same date from 23 ‘nonparthenocarpic’ plants. From the two DNA samples, in the Technion, (The Life Sciences and Engineering Infrastructure Center) Haifa, Israel, two sequencing libraries, one designated ‘2012 library’ and the other ‘NP (nonparthenocarpic) library’, were prepared and sequenced using 100‐bp paired end reads on an Illumina HiSeq 2000 platform. Bioinformatics analysis was performed following Bolger *et al*. (2014a,b), Li and Durbin (2009), Li *et al*. (2009) and Schneeberger *et al*. (2009), as detailed in Data S1.

### Construction of a CRISPR/Cas9 knockout plasmid and tomato transformation

The CRISPR/Cas9 construct was designed to create a deletion/insertion after 212 bp of the *Solyc01g093960* coding sequence (predicted exon 2, see Figure 2b). The 20‐bp target sequence was chosen to be followed by protospacer adjacent motif (PAM), the requisite binding site for Cas9, TGG (depicted in Figure 2b). The selected sgRNA was amplified using the primers: SalI‐gRNA‐F: AGA*gtcgac*ATAGCGATTGAGGATTAAGGCAACAACGTGTTTTAGAGCTAGAAATAGCAAG and HindIII‐gRNA‐R: TAAGCT*aagctt*CGATCTAAAAAAAGCACCGACT (the added restriction sites are presented in italics lowercase letters, and the specific target sequence (cRNA) is underlined in the SalI‐gRNA‐F primer sequence). The PCR product was restricted and cloned into the pRCS binary vector SalI‐HindIII sites as previously described (Chandrasekaran *et al*., 2016). The binary vector was transformed to *Agrobacterium tumefaciens* strain EHA105, which served to transform the indeterminate tomato line MP‐1 as previously described (Barg *et al*., 1997).

### Detection of CRISPR/Cas9‐induced mutations

The *Solyc01g093960* gRNA target site was designed to include AclI restriction enzyme site overlapping three bp upstream from the PAM (see Figure 2a), the predicted cut site of the Cas9 nuclease, so that DNA double‐strand break repair could disrupt the restriction site. *R*_o_ plants were screened for the presence of chimeric section as detailed in Data S1, and the same procedure was used to genotype *R*_1_ progenies. The presence of the Cas9/sgRNA cassette in the progenies was PCR‐tested.

### Quantitative RT‐PCR analysis of Sl*AGL*6

Analysis was performed on total RNA samples extracted from developing ovaries and young fruit collected from line M82 plants, as earlier described (Damodharan *et al*., 2016).

### Pollen viability assay

Freshly harvested pollen grains were incubated in germination solution (Firon *et al*., 2012) for 18 h, at 24 °C, and examined under light microscope.

All primers used in this study are listed in Table S1.

### Statistical analysis

ANOVA statistical analyses were performed with SIGMASTAT 2.0 program. (http://en.softonic.com/s/sigma-stat-version-2/).

## Supporting information


**Figure S1.** Parthenocarpic fruits of *R*_1_ plants homozygous for three differently CRISPR/Cas9 mutated Sl*AGL6* alleles.

**Figure S2.** Manifestation of the 2012 mutation in a larger fruit background.

**Figure S3.** Daily maximum and minimum temperatures experienced by the plants in the various experiments.

**Figure S4.** Reported modes of expression of Sl*AGL6*.

**Table S1.** Primers used in this study.

**Data S1.** Methods.
