# Supplementary material for: Tomato facultative parthenocarpy results from Sl AGAMOUS‐LIKE 6 loss of function
Source: Plant Biotechnol J. 2016 Dec 27;15(5):634–47. doi: 10.1111/pbi.12662 (PMC5399002; doi:10.1111/pbi.12662)
Supplement: Supplementary file 1 — Figure S1. Parthenocarpic fruits of R 1 plants homozygous for three differently CRISPR/Cas9 mutated SlAGL6 alleles. [file PBI-15-634-s006.pptx]

## Slide 1
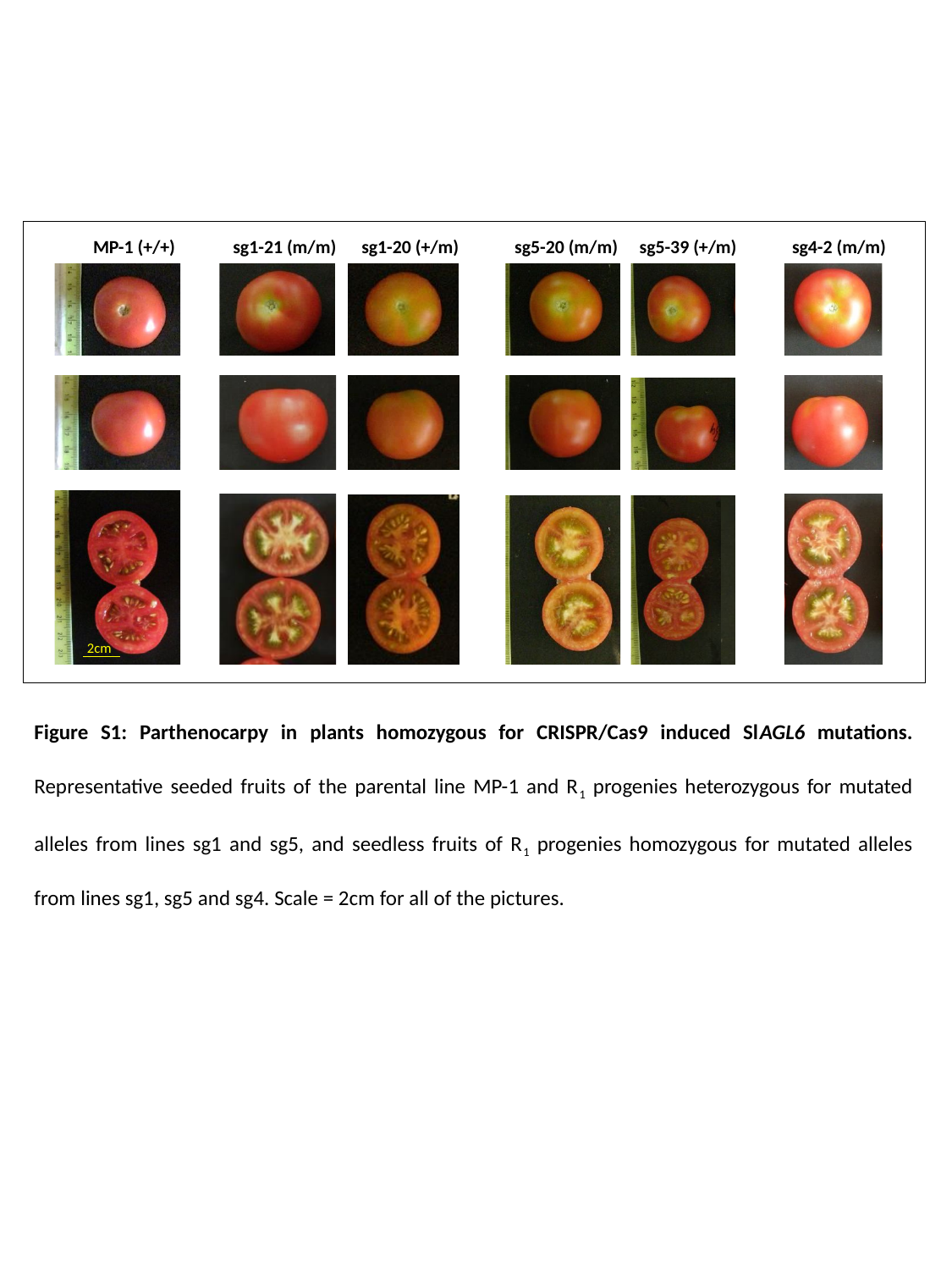

sg1-21 (m/m)
sg1-20 (+/m)
MP-1 (+/+)
sg5-20 (m/m)
sg5-39 (+/m)
sg4-2 (m/m)
2cm
Figure S1: Parthenocarpy in plants homozygous for CRISPR/Cas9 induced SlAGL6 mutations. Representative seeded fruits of the parental line MP-1 and R1 progenies heterozygous for mutated alleles from lines sg1 and sg5, and seedless fruits of R1 progenies homozygous for mutated alleles from lines sg1, sg5 and sg4. Scale = 2cm for all of the pictures.
