# Supplementary material for: Tomato facultative parthenocarpy results from Sl AGAMOUS‐LIKE 6 loss of function
Source: Plant Biotechnol J. 2016 Dec 27;15(5):634–47. doi: 10.1111/pbi.12662 (PMC5399002; doi:10.1111/pbi.12662)
Supplement: Supplementary file 2 — Figure S2. Manifestation of the 2012 mutation in a larger fruit background. [file PBI-15-634-s005.pptx]

## Slide 1
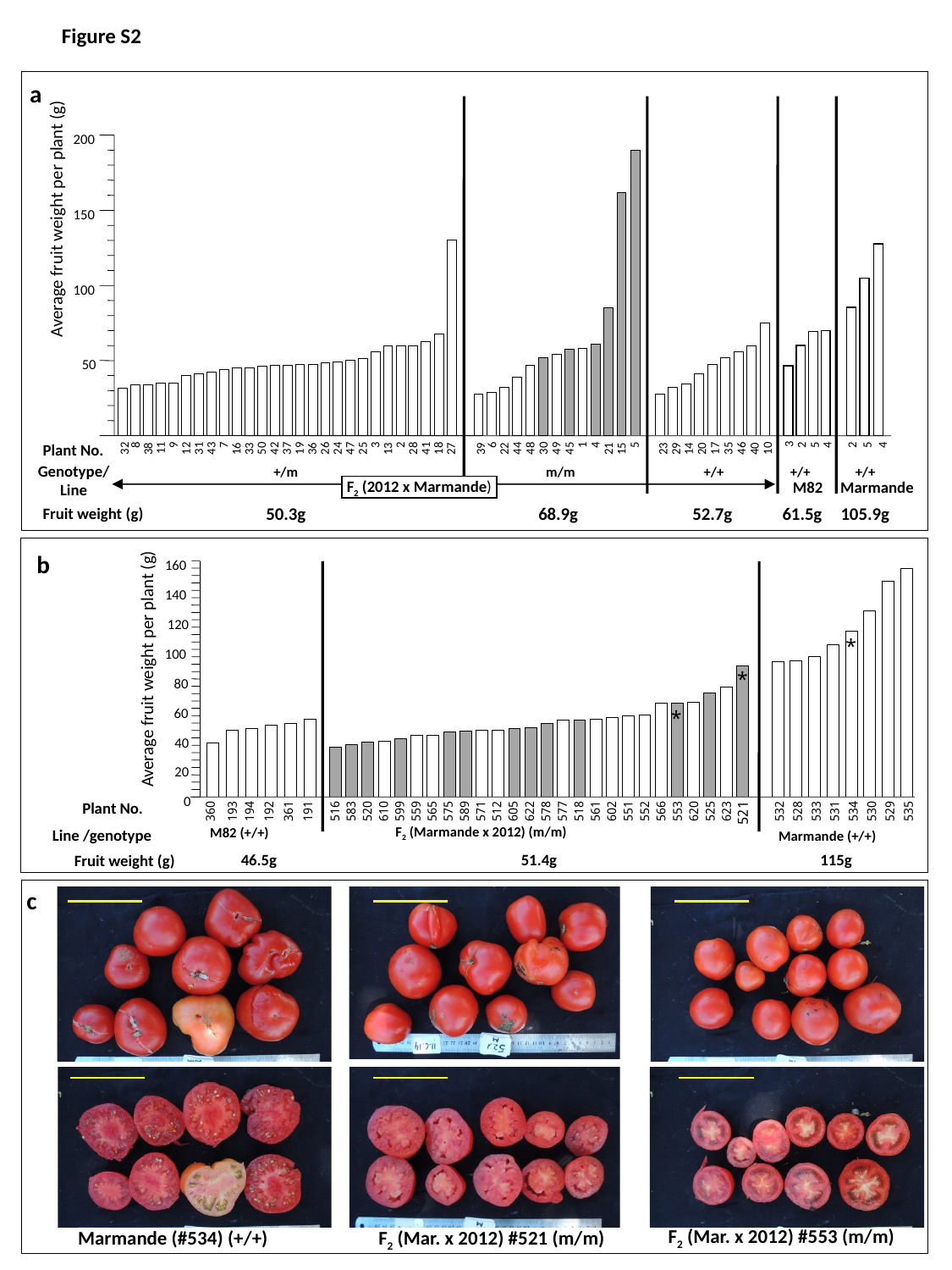

Figure S2
a
200
150
100
50
Average fruit weight per plant (g)
3
8
9
7
3
2
6
1
4
5
2
5
4
2
5
4
Plant No.
11
32
38
12
31
43
16
33
50
42
37
19
36
26
24
47
25
13
28
41
18
27
39
22
44
48
30
49
45
21
15
23
29
14
20
17
35
46
40
10
Genotype/
Line
+/m
m/m
+/+
+/+
+/+
F2 (2012 x Marmande)
M82
Marmande
50.3g
68.9g
52.7g
61.5g
105.9g
Fruit weight (g)
b
160
140
120
*
100
Average fruit weight per plant (g)
*
80
60
*
40
20
0
Plant No.
360
193
194
192
361
191
516
583
520
610
599
559
565
575
589
571
512
605
622
578
577
518
561
602
551
552
566
553
620
525
623
532
528
533
531
534
530
529
535
521
M82 (+/+)
F2 (Marmande x 2012) (m/m)
Line /genotype
Marmande (+/+)
46.5g
51.4g
115g
Fruit weight (g)
c
Marmande (#534) (+/+)
F2 (Mar. x 2012) #553 (m/m)
F2 (Mar. x 2012) #521 (m/m)

## Slide 2
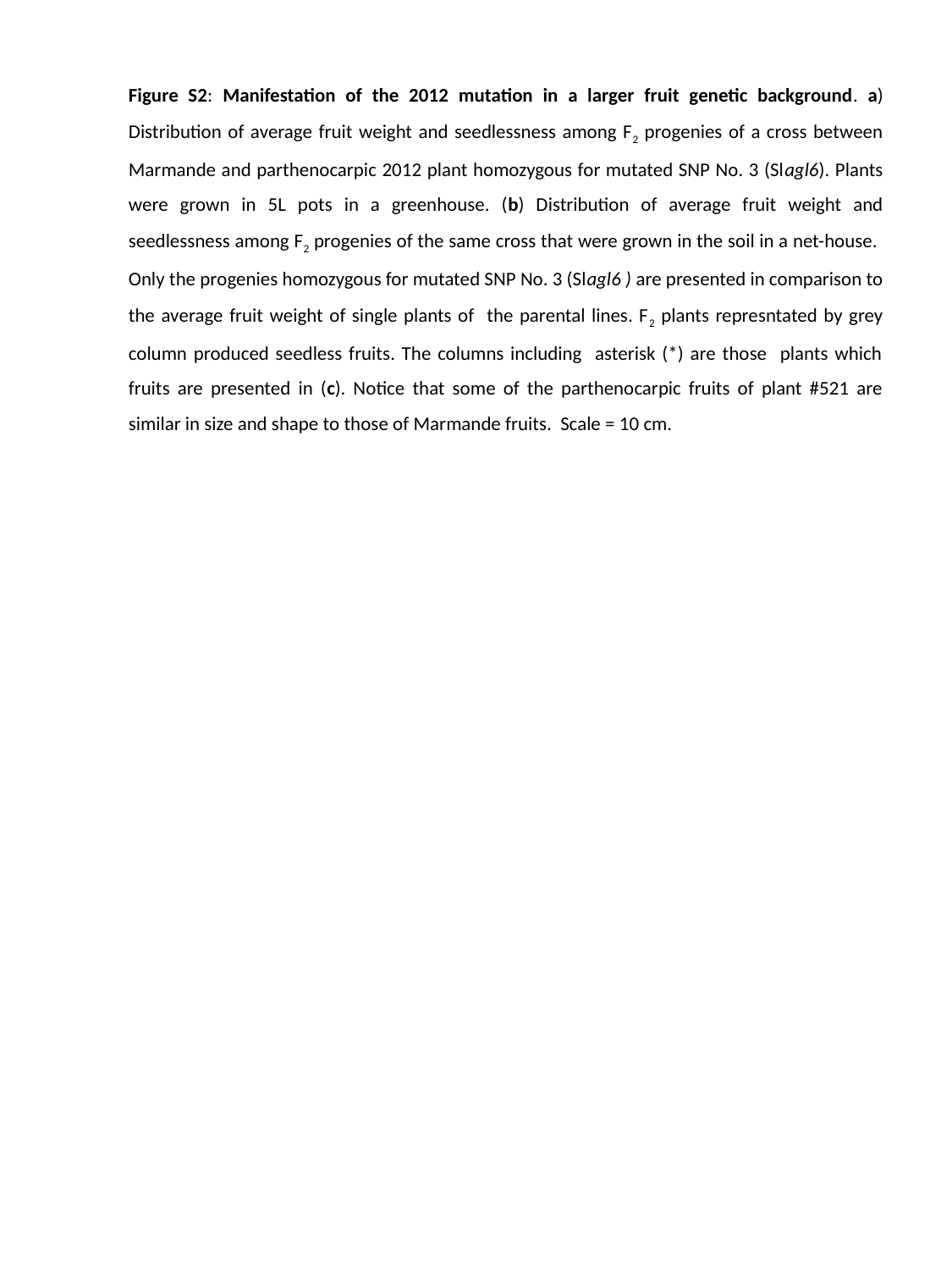

Figure S2: Manifestation of the 2012 mutation in a larger fruit genetic background. a) Distribution of average fruit weight and seedlessness among F2 progenies of a cross between Marmande and parthenocarpic 2012 plant homozygous for mutated SNP No. 3 (Slagl6). Plants were grown in 5L pots in a greenhouse. (b) Distribution of average fruit weight and seedlessness among F2 progenies of the same cross that were grown in the soil in a net-house. Only the progenies homozygous for mutated SNP No. 3 (Slagl6 ) are presented in comparison to the average fruit weight of single plants of the parental lines. F2 plants represntated by grey column produced seedless fruits. The columns including asterisk (*) are those plants which fruits are presented in (c). Notice that some of the parthenocarpic fruits of plant #521 are similar in size and shape to those of Marmande fruits. Scale = 10 cm.
