# Supplementary material for: Tomato facultative parthenocarpy results from Sl AGAMOUS‐LIKE 6 loss of function
Source: Plant Biotechnol J. 2016 Dec 27;15(5):634–47. doi: 10.1111/pbi.12662 (PMC5399002; doi:10.1111/pbi.12662)
Supplement: Supplementary file 3 — Figure S3. Daily maximum and minimum temperatures experienced by the plants in the various experiments. [file PBI-15-634-s004.pptx]

## Slide 1
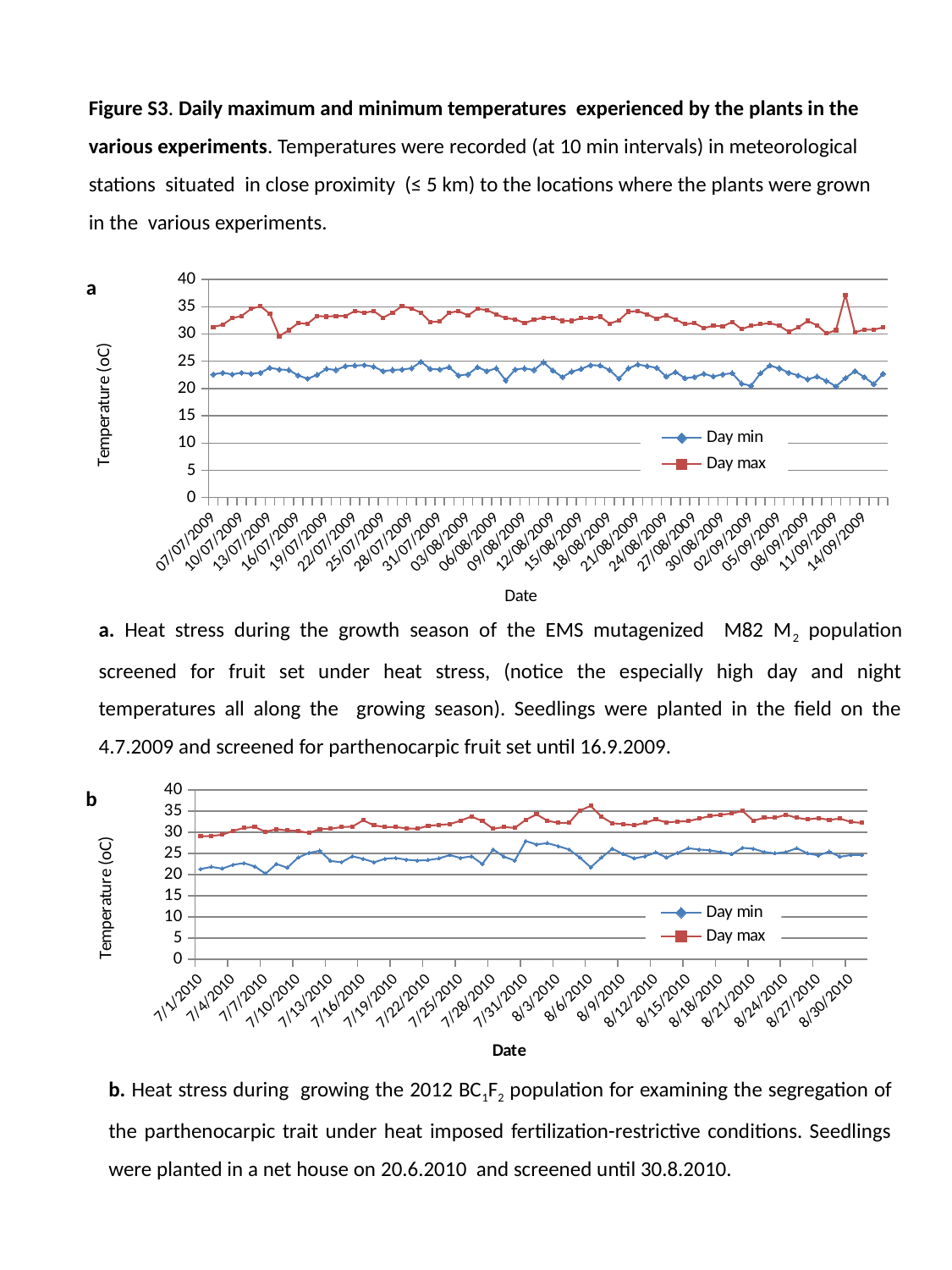

Figure S3. Daily maximum and minimum temperatures experienced by the plants in the various experiments. Temperatures were recorded (at 10 min intervals) in meteorological stations situated in close proximity (≤ 5 km) to the locations where the plants were grown in the various experiments.
### Chart
| Category | Day min | Day max |
|---|---|---|
| 07/07/2009 | 22.6 | 31.3 |
| 08/07/2009 | 22.9 | 31.7 |
| 09/07/2009 | 22.6 | 32.9 |
| 10/07/2009 | 22.9 | 33.3 |
| 11/07/2009 | 22.7 | 34.6 |
| 12/07/2009 | 22.9 | 35.1 |
| 13/07/2009 | 23.8 | 33.7 |
| 14/07/2009 | 23.5 | 29.6 |
| 15/07/2009 | 23.4 | 30.7 |
| 16/07/2009 | 22.4 | 32.0 |
| 17/07/2009 | 21.8 | 31.9 |
| 18/07/2009 | 22.5 | 33.3 |
| 19/07/2009 | 23.6 | 33.2 |
| 20/07/2009 | 23.4 | 33.3 |
| 21/07/2009 | 24.1 | 33.3 |
| 22/07/2009 | 24.2 | 34.2 |
| 23/07/2009 | 24.3 | 33.9 |
| 24/07/2009 | 24.0 | 34.2 |
| 25/07/2009 | 23.2 | 33.0 |
| 26/07/2009 | 23.4 | 33.9 |
| 27/07/2009 | 23.5 | 35.1 |
| 28/07/2009 | 23.7 | 34.7 |
| 29/07/2009 | 24.9 | 33.9 |
| 30/07/2009 | 23.6 | 32.2 |
| 31/07/2009 | 23.5 | 32.3 |
| 01/08/2009 | 23.9 | 33.9 |
| 02/08/2009 | 22.4 | 34.2 |
| 03/08/2009 | 22.6 | 33.4 |
| 04/08/2009 | 23.9 | 34.6 |
| 05/08/2009 | 23.2 | 34.4 |
| 06/08/2009 | 23.7 | 33.6 |
| 07/08/2009 | 21.5 | 32.9 |
| 08/08/2009 | 23.5 | 32.7 |
| 09/08/2009 | 23.7 | 32.0 |
| 10/08/2009 | 23.4 | 32.6 |
| 11/08/2009 | 24.8 | 33.0 |
| 12/08/2009 | 23.3 | 33.0 |
| 13/08/2009 | 22.1 | 32.4 |
| 14/08/2009 | 23.1 | 32.4 |
| 15/08/2009 | 23.6 | 32.9 |
| 16/08/2009 | 24.3 | 32.9 |
| 17/08/2009 | 24.2 | 33.2 |
| 18/08/2009 | 23.4 | 31.9 |
| 19/08/2009 | 21.8 | 32.5 |
| 20/08/2009 | 23.7 | 34.1 |
| 21/08/2009 | 24.4 | 34.2 |
| 22/08/2009 | 24.1 | 33.6 |
| 23/08/2009 | 23.8 | 32.8 |
| 24/08/2009 | 22.2 | 33.4 |
| 25/08/2009 | 23.0 | 32.7 |
| 26/08/2009 | 21.9 | 31.8 |
| 27/08/2009 | 22.1 | 32.0 |
| 28/08/2009 | 22.7 | 31.1 |
| 29/08/2009 | 22.2 | 31.5 |
| 30/08/2009 | 22.6 | 31.4 |
| 31/08/2009 | 22.8 | 32.2 |
| 01/09/2009 | 20.9 | 30.9 |
| 02/09/2009 | 20.5 | 31.5 |
| 03/09/2009 | 22.8 | 31.8 |
| 04/09/2009 | 24.2 | 32.0 |
| 05/09/2009 | 23.7 | 31.5 |
| 06/09/2009 | 22.9 | 30.4 |
| 07/09/2009 | 22.4 | 31.2 |
| 08/09/2009 | 21.7 | 32.4 |
| 09/09/2009 | 22.2 | 31.6 |
| 10/09/2009 | 21.4 | 30.1 |
| 11/09/2009 | 20.4 | 30.7 |
| 12/09/2009 | 21.9 | 37.2 |
| 13/09/2009 | 23.2 | 30.3 |
| 14/09/2009 | 22.1 | 30.8 |
| 15/09/2009 | 20.8 | 30.8 |
| 16/09/2009 | 22.7 | 31.2 |a
a. Heat stress during the growth season of the EMS mutagenized M82 M2 population screened for fruit set under heat stress, (notice the especially high day and night temperatures all along the growing season). Seedlings were planted in the field on the 4.7.2009 and screened for parthenocarpic fruit set until 16.9.2009.
### Chart
| Category | Day min | Day max |
|---|---|---|
| 40360 | 21.3 | 29.0 |
| 40361 | 21.8 | 29.1 |
| 40362 | 21.4 | 29.4 |
| 40363 | 22.3 | 30.3 |
| 40364 | 22.7 | 31.0 |
| 40365 | 21.9 | 31.2 |
| 40366 | 20.2 | 30.1 |
| 40367 | 22.5 | 30.7 |
| 40368 | 21.6 | 30.4 |
| 40369 | 24.0 | 30.3 |
| 40370 | 25.1 | 29.8 |
| 40371 | 25.6 | 30.7 |
| 40372 | 23.2 | 30.8 |
| 40373 | 22.9 | 31.2 |
| 40374 | 24.3 | 31.3 |
| 40375 | 23.7 | 32.8 |
| 40376 | 22.9 | 31.6 |
| 40377 | 23.7 | 31.2 |
| 40378 | 23.9 | 31.2 |
| 40379 | 23.5 | 30.9 |
| 40380 | 23.3 | 30.8 |
| 40381 | 23.4 | 31.5 |
| 40382 | 23.8 | 31.7 |
| 40383 | 24.6 | 31.9 |
| 40384 | 23.9 | 32.7 |
| 40385 | 24.3 | 33.7 |
| 40386 | 22.5 | 32.6 |
| 40387 | 25.9 | 30.8 |
| 40388 | 24.2 | 31.2 |
| 40389 | 23.3 | 31.0 |
| 40390 | 27.9 | 32.9 |
| 40391 | 27.1 | 34.2 |
| 40392 | 27.4 | 32.7 |
| 40393 | 26.7 | 32.2 |
| 40394 | 25.9 | 32.2 |
| 40395 | 24.0 | 35.1 |
| 40396 | 21.7 | 36.2 |
| 40397 | 24.0 | 33.6 |
| 40398 | 26.1 | 32.1 |
| 40399 | 24.8 | 31.9 |
| 40400 | 23.8 | 31.6 |
| 40401 | 24.3 | 32.2 |
| 40402 | 25.2 | 33.0 |
| 40403 | 24.0 | 32.3 |
| 40404 | 25.1 | 32.5 |
| 40405 | 26.2 | 32.6 |
| 40406 | 25.9 | 33.2 |
| 40407 | 25.7 | 33.8 |
| 40408 | 25.3 | 34.1 |
| 40409 | 24.8 | 34.4 |
| 40410 | 26.3 | 35.0 |
| 40411 | 26.1 | 32.7 |
| 40412 | 25.3 | 33.4 |
| 40413 | 25.0 | 33.4 |
| 40414 | 25.3 | 34.1 |
| 40415 | 26.2 | 33.4 |
| 40416 | 25.0 | 33.0 |
| 40417 | 24.5 | 33.3 |
| 40418 | 25.4 | 32.9 |
| 40419 | 24.2 | 33.2 |
| 40420 | 24.6 | 32.4 |
| 40421 | 24.6 | 32.2 |b
b. Heat stress during growing the 2012 BC1F2 population for examining the segregation of the parthenocarpic trait under heat imposed fertilization-restrictive conditions. Seedlings were planted in a net house on 20.6.2010 and screened until 30.8.2010.

## Slide 2
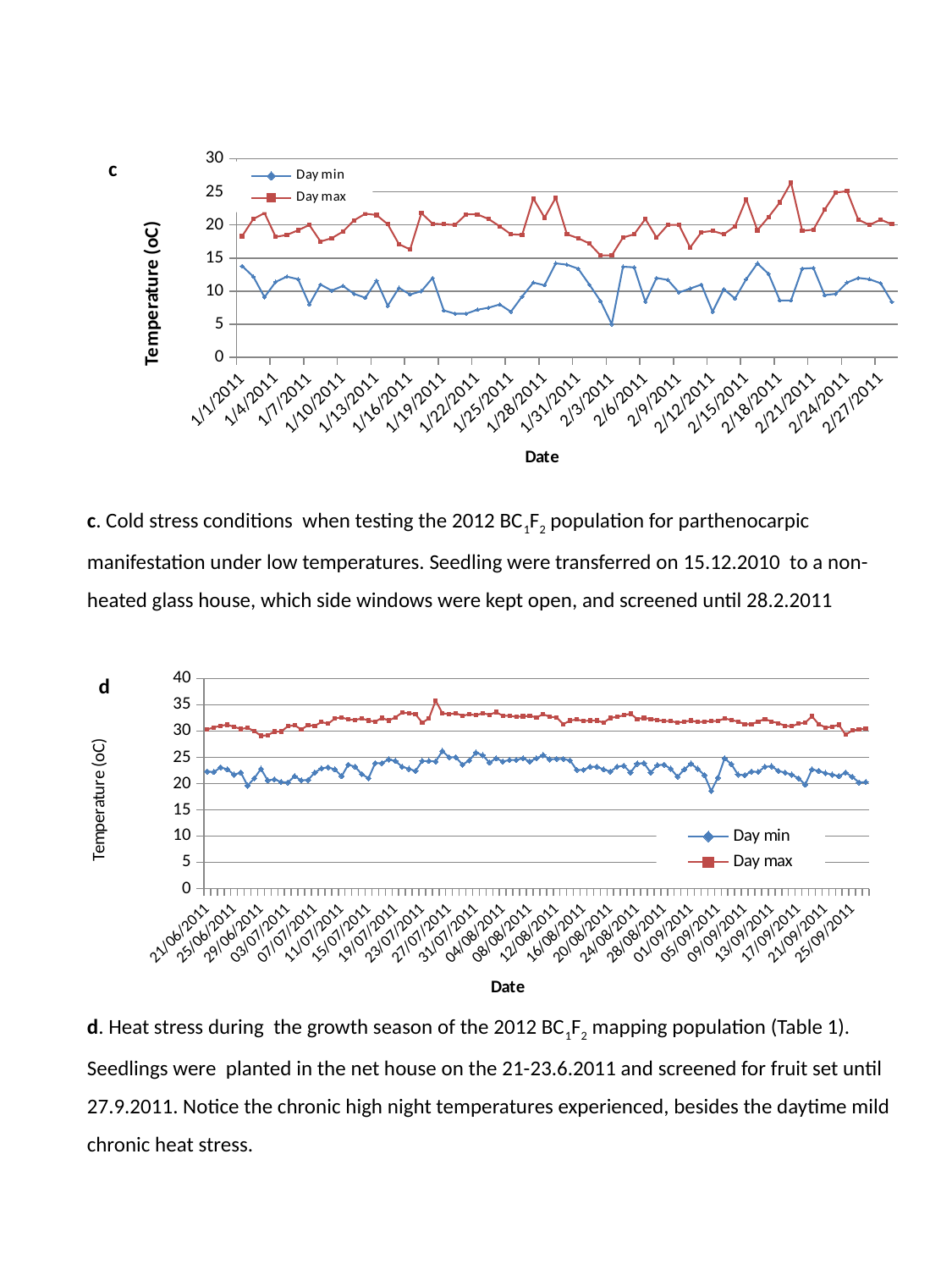

### Chart
| Category | Day min | Day max |
|---|---|---|
| 40544 | 13.8 | 18.3 |
| 40545 | 12.2 | 20.9 |
| 40546 | 9.1 | 21.8 |
| 40547 | 11.4 | 18.2 |
| 40548 | 12.2 | 18.5 |
| 40549 | 11.8 | 19.2 |
| 40550 | 8.0 | 20.0 |
| 40551 | 11.0 | 17.5 |
| 40552 | 10.1 | 18.0 |
| 40553 | 10.8 | 19.0 |
| 40554 | 9.6 | 20.7 |
| 40555 | 9.0 | 21.7 |
| 40556 | 11.6 | 21.5 |
| 40557 | 7.8 | 20.1 |
| 40558 | 10.5 | 17.1 |
| 40559 | 9.5 | 16.3 |
| 40560 | 10.0 | 21.8 |
| 40561 | 12.0 | 20.2 |
| 40562 | 7.1 | 20.1 |
| 40563 | 6.6 | 20.0 |
| 40564 | 6.6 | 21.6 |
| 40565 | 7.2 | 21.6 |
| 40566 | 7.5 | 20.9 |
| 40567 | 8.0 | 19.8 |
| 40568 | 6.9 | 18.6 |
| 40569 | 9.2 | 18.5 |
| 40570 | 11.3 | 24.0 |
| 40571 | 10.9 | 21.1 |
| 40572 | 14.2 | 24.1 |
| 40573 | 14.0 | 18.6 |
| 40574 | 13.4 | 18.0 |
| 40575 | 11.0 | 17.2 |
| 40576 | 8.5 | 15.4 |
| 40577 | 5.0 | 15.4 |
| 40578 | 13.7 | 18.1 |
| 40579 | 13.6 | 18.6 |
| 40580 | 8.4 | 20.9 |
| 40581 | 12.0 | 18.1 |
| 40582 | 11.7 | 20.0 |
| 40583 | 9.8 | 20.0 |
| 40584 | 10.4 | 16.6 |
| 40585 | 11.0 | 18.9 |
| 40586 | 6.9 | 19.1 |
| 40587 | 10.3 | 18.6 |
| 40588 | 8.9 | 19.8 |
| 40589 | 11.8 | 23.9 |
| 40590 | 14.2 | 19.2 |
| 40591 | 12.6 | 21.2 |
| 40592 | 8.6 | 23.4 |
| 40593 | 8.6 | 26.4 |
| 40594 | 13.4 | 19.1 |
| 40595 | 13.5 | 19.3 |
| 40596 | 9.4 | 22.3 |
| 40597 | 9.6 | 24.9 |
| 40598 | 11.3 | 25.1 |
| 40599 | 12.0 | 20.8 |
| 40600 | 11.8 | 20.0 |
| 40601 | 11.2 | 20.8 |
| 40602 | 8.4 | 20.1 |c
c. Cold stress conditions when testing the 2012 BC1F2 population for parthenocarpic manifestation under low temperatures. Seedling were transferred on 15.12.2010 to a non-heated glass house, which side windows were kept open, and screened until 28.2.2011
### Chart
| Category | Day min | Day max |
|---|---|---|
| 21/06/2011 | 22.3 | 30.3 |
| 22/06/2011 | 22.2 | 30.7 |
| 23/06/2011 | 23.1 | 31.0 |
| 24/06/2011 | 22.7 | 31.2 |
| 25/06/2011 | 21.7 | 30.8 |
| 26/06/2011 | 22.1 | 30.4 |
| 27/06/2011 | 19.6 | 30.6 |
| 28/06/2011 | 21.0 | 30.0 |
| 29/06/2011 | 22.8 | 29.1 |
| 30/06/2011 | 20.6 | 29.2 |
| 01/07/2011 | 20.8 | 29.9 |
| 02/07/2011 | 20.3 | 29.9 |
| 03/07/2011 | 20.2 | 30.9 |
| 04/07/2011 | 21.4 | 31.1 |
| 05/07/2011 | 20.6 | 30.3 |
| 06/07/2011 | 20.7 | 31.1 |
| 07/07/2011 | 22.1 | 31.0 |
| 08/07/2011 | 22.9 | 31.7 |
| 09/07/2011 | 23.1 | 31.4 |
| 10/07/2011 | 22.7 | 32.4 |
| 11/07/2011 | 21.4 | 32.6 |
| 12/07/2011 | 23.6 | 32.2 |
| 13/07/2011 | 23.2 | 32.1 |
| 14/07/2011 | 21.8 | 32.4 |
| 15/07/2011 | 21.0 | 32.0 |
| 16/07/2011 | 23.9 | 31.8 |
| 17/07/2011 | 23.9 | 32.5 |
| 18/07/2011 | 24.6 | 32.0 |
| 19/07/2011 | 24.3 | 32.6 |
| 20/07/2011 | 23.2 | 33.5 |
| 21/07/2011 | 22.8 | 33.4 |
| 22/07/2011 | 22.4 | 33.2 |
| 23/07/2011 | 24.3 | 31.6 |
| 24/07/2011 | 24.3 | 32.4 |
| 25/07/2011 | 24.2 | 35.8 |
| 26/07/2011 | 26.2 | 33.4 |
| 27/07/2011 | 25.0 | 33.2 |
| 28/07/2011 | 25.0 | 33.4 |
| 29/07/2011 | 23.6 | 32.9 |
| 30/07/2011 | 24.4 | 33.2 |
| 31/07/2011 | 25.9 | 33.0 |
| 01/08/2011 | 25.4 | 33.4 |
| 02/08/2011 | 24.0 | 33.1 |
| 03/08/2011 | 24.8 | 33.6 |
| 04/08/2011 | 24.2 | 32.9 |
| 05/08/2011 | 24.5 | 32.9 |
| 06/08/2011 | 24.5 | 32.7 |
| 07/08/2011 | 24.8 | 32.8 |
| 08/08/2011 | 24.2 | 32.9 |
| 09/08/2011 | 24.8 | 32.6 |
| 10/08/2011 | 25.4 | 33.2 |
| 11/08/2011 | 24.6 | 32.7 |
| 12/08/2011 | 24.7 | 32.6 |
| 13/08/2011 | 24.7 | 31.3 |
| 14/08/2011 | 24.4 | 32.0 |
| 15/08/2011 | 22.6 | 32.2 |
| 16/08/2011 | 22.6 | 31.9 |
| 17/08/2011 | 23.2 | 32.0 |
| 18/08/2011 | 23.2 | 32.0 |
| 19/08/2011 | 22.7 | 31.6 |
| 20/08/2011 | 22.3 | 32.5 |
| 21/08/2011 | 23.2 | 32.7 |
| 22/08/2011 | 23.4 | 33.0 |
| 23/08/2011 | 22.1 | 33.3 |
| 24/08/2011 | 23.8 | 32.3 |
| 25/08/2011 | 23.9 | 32.5 |
| 26/08/2011 | 22.1 | 32.3 |
| 27/08/2011 | 23.5 | 32.1 |
| 28/08/2011 | 23.6 | 31.9 |
| 29/08/2011 | 22.8 | 31.9 |
| 30/08/2011 | 21.3 | 31.6 |
| 31/08/2011 | 22.7 | 31.8 |
| 01/09/2011 | 23.8 | 32.0 |
| 02/09/2011 | 22.8 | 31.7 |
| 03/09/2011 | 21.6 | 31.8 |
| 04/09/2011 | 18.6 | 31.9 |
| 05/09/2011 | 21.1 | 31.9 |
| 06/09/2011 | 24.8 | 32.4 |
| 07/09/2011 | 23.7 | 32.1 |
| 08/09/2011 | 21.7 | 31.8 |
| 09/09/2011 | 21.6 | 31.3 |
| 10/09/2011 | 22.3 | 31.3 |
| 11/09/2011 | 22.2 | 31.8 |
| 12/09/2011 | 23.2 | 32.3 |
| 13/09/2011 | 23.3 | 31.8 |
| 14/09/2011 | 22.4 | 31.5 |
| 15/09/2011 | 22.1 | 31.0 |
| 16/09/2011 | 21.7 | 30.9 |
| 17/09/2011 | 21.0 | 31.4 |
| 18/09/2011 | 19.8 | 31.6 |
| 19/09/2011 | 22.7 | 32.8 |
| 20/09/2011 | 22.4 | 31.3 |
| 21/09/2011 | 22.0 | 30.7 |
| 22/09/2011 | 21.7 | 30.8 |
| 23/09/2011 | 21.4 | 31.2 |
| 24/09/2011 | 22.1 | 29.3 |
| 25/09/2011 | 21.3 | 30.1 |
| 26/09/2011 | 20.2 | 30.3 |
| 27/09/2011 | 20.3 | 30.5 |d
d. Heat stress during the growth season of the 2012 BC1F2 mapping population (Table 1). Seedlings were planted in the net house on the 21-23.6.2011 and screened for fruit set until 27.9.2011. Notice the chronic high night temperatures experienced, besides the daytime mild chronic heat stress.

## Slide 3
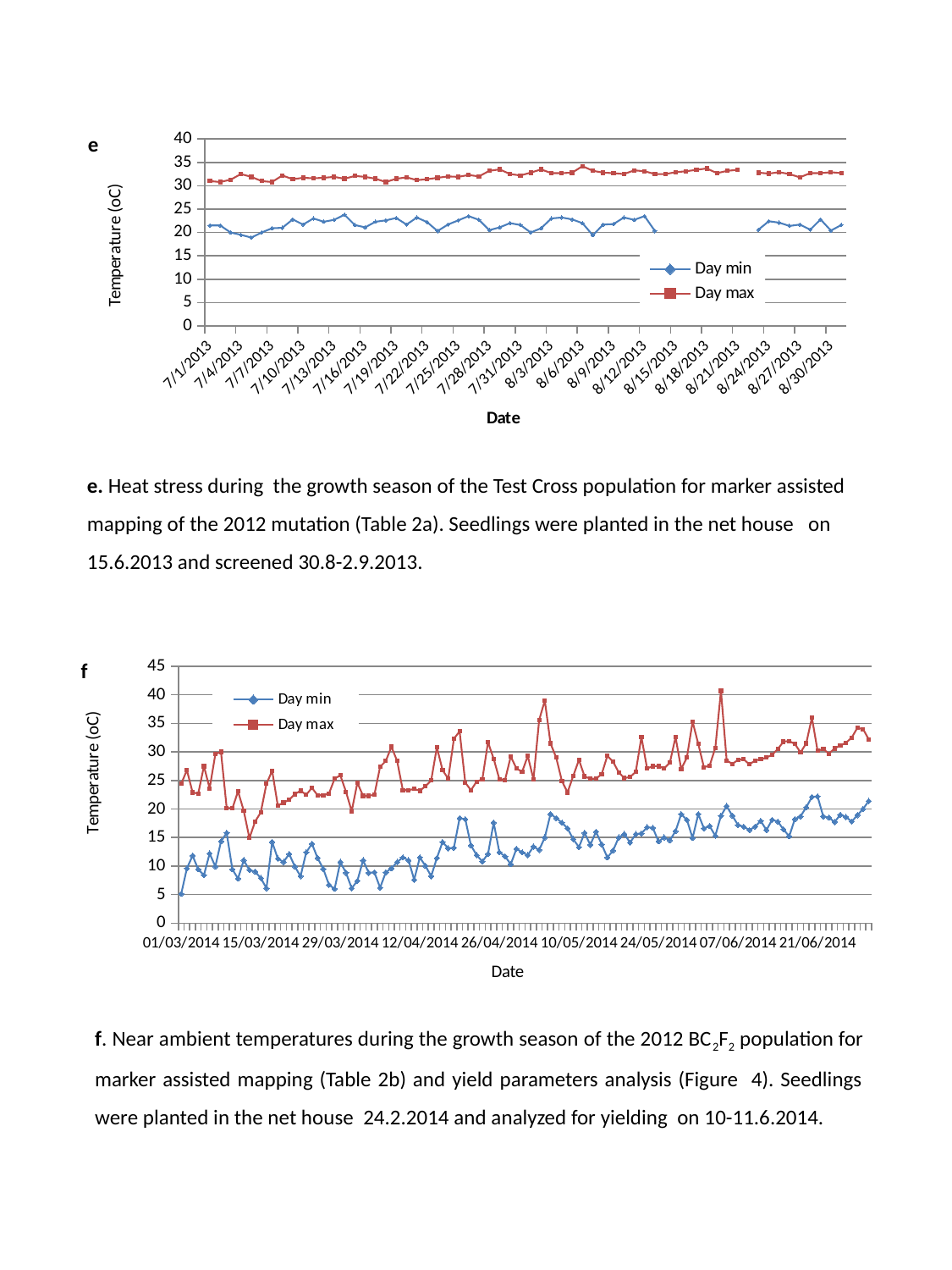

### Chart
| Category | Day min | Day max |
|---|---|---|
| 41456 | 21.5 | 31.0 |
| 41457 | 21.5 | 30.8 |
| 41458 | 20.0 | 31.3 |
| 41459 | 19.5 | 32.5 |
| 41460 | 18.9 | 31.9 |
| 41461 | 20.0 | 31.0 |
| 41462 | 20.9 | 30.8 |
| 41463 | 21.0 | 32.2 |
| 41464 | 22.8 | 31.4 |
| 41465 | 21.7 | 31.7 |
| 41466 | 23.0 | 31.6 |
| 41467 | 22.3 | 31.7 |
| 41468 | 22.7 | 31.9 |
| 41469 | 23.8 | 31.5 |
| 41470 | 21.6 | 32.1 |
| 41471 | 21.1 | 31.9 |
| 41472 | 22.3 | 31.5 |
| 41473 | 22.6 | 30.8 |
| 41474 | 23.1 | 31.5 |
| 41475 | 21.7 | 31.8 |
| 41476 | 23.2 | 31.2 |
| 41477 | 22.2 | 31.4 |
| 41478 | 20.3 | 31.7 |
| 41479 | 21.7 | 32.0 |
| 41480 | 22.6 | 31.9 |
| 41481 | 23.5 | 32.3 |
| 41482 | 22.7 | 32.0 |
| 41483 | 20.5 | 33.2 |
| 41484 | 21.1 | 33.5 |
| 41485 | 22.0 | 32.5 |
| 41486 | 21.6 | 32.2 |
| 41487 | 20.0 | 32.8 |
| 41488 | 20.9 | 33.5 |
| 41489 | 23.0 | 32.7 |
| 41490 | 23.2 | 32.7 |
| 41491 | 22.8 | 32.8 |
| 41492 | 22.0 | 34.2 |
| 41493 | 19.4 | 33.2 |
| 41494 | 21.7 | 32.8 |
| 41495 | 21.8 | 32.7 |
| 41496 | 23.2 | 32.5 |
| 41497 | 22.7 | 33.3 |
| 41498 | 23.5 | 33.1 |
| 41499 | 20.3 | 32.5 |
| 41500 | None | 32.5 |
| 41501 | None | 32.9 |
| 41502 | None | 33.1 |
| 41503 | None | 33.4 |
| 41504 | None | 33.7 |
| 41505 | None | 32.7 |
| 41506 | None | 33.2 |
| 41507 | None | 33.4 |
| 41508 | None | None |
| 41509 | 20.6 | 32.8 |
| 41510 | 22.4 | 32.6 |
| 41511 | 22.1 | 32.9 |
| 41512 | 21.4 | 32.5 |
| 41513 | 21.7 | 31.8 |
| 41514 | 20.6 | 32.7 |
| 41515 | 22.8 | 32.7 |
| 41516 | 20.4 | 32.9 |
| 41517 | 21.6 | 32.7 |e
e. Heat stress during the growth season of the Test Cross population for marker assisted mapping of the 2012 mutation (Table 2a). Seedlings were planted in the net house on 15.6.2013 and screened 30.8-2.9.2013.
### Chart
| Category | Day min | Day max |
|---|---|---|
| 01/03/2014 | 5.1 | 24.4 |
| 02/03/2014 | 9.6 | 26.8 |
| 03/03/2014 | 11.8 | 22.9 |
| 04/03/2014 | 9.4 | 22.7 |
| 05/03/2014 | 8.4 | 27.5 |
| 06/03/2014 | 12.2 | 23.6 |
| 07/03/2014 | 9.9 | 29.6 |
| 08/03/2014 | 14.3 | 30.0 |
| 09/03/2014 | 15.8 | 20.2 |
| 10/03/2014 | 9.4 | 20.1 |
| 11/03/2014 | 7.8 | 23.1 |
| 12/03/2014 | 11.0 | 19.7 |
| 13/03/2014 | 9.3 | 14.9 |
| 14/03/2014 | 9.0 | 17.8 |
| 15/03/2014 | 7.9 | 19.4 |
| 16/03/2014 | 6.1 | 24.4 |
| 17/03/2014 | 14.2 | 26.7 |
| 18/03/2014 | 11.3 | 20.6 |
| 19/03/2014 | 10.7 | 21.1 |
| 20/03/2014 | 12.1 | 21.6 |
| 21/03/2014 | 9.9 | 22.6 |
| 22/03/2014 | 8.2 | 23.2 |
| 23/03/2014 | 12.4 | 22.5 |
| 24/03/2014 | 13.9 | 23.7 |
| 25/03/2014 | 11.4 | 22.4 |
| 26/03/2014 | 9.5 | 22.4 |
| 27/03/2014 | 6.7 | 22.7 |
| 28/03/2014 | 6.0 | 25.3 |
| 29/03/2014 | 10.7 | 25.9 |
| 30/03/2014 | 8.8 | 23.0 |
| 31/03/2014 | 6.1 | 19.6 |
| 01/04/2014 | 7.4 | 24.6 |
| 02/04/2014 | 11.0 | 22.3 |
| 03/04/2014 | 8.8 | 22.3 |
| 04/04/2014 | 8.9 | 22.5 |
| 05/04/2014 | 6.2 | 27.4 |
| 06/04/2014 | 8.9 | 28.4 |
| 07/04/2014 | 9.6 | 30.9 |
| 08/04/2014 | 10.7 | 28.4 |
| 09/04/2014 | 11.5 | 23.3 |
| 10/04/2014 | 11.0 | 23.3 |
| 11/04/2014 | 7.6 | 23.5 |
| 12/04/2014 | 11.5 | 23.2 |
| 13/04/2014 | 10.0 | 24.0 |
| 14/04/2014 | 8.2 | 25.0 |
| 15/04/2014 | 11.4 | 30.8 |
| 16/04/2014 | 14.2 | 26.8 |
| 17/04/2014 | 13.1 | 25.3 |
| 18/04/2014 | 13.2 | 32.3 |
| 19/04/2014 | 18.4 | 33.6 |
| 20/04/2014 | 18.2 | 24.6 |
| 21/04/2014 | 13.6 | 23.3 |
| 22/04/2014 | 11.9 | 24.8 |
| 23/04/2014 | 10.8 | 25.2 |
| 24/04/2014 | 12.1 | 31.7 |
| 25/04/2014 | 17.6 | 28.8 |
| 26/04/2014 | 12.4 | 25.2 |
| 27/04/2014 | 11.7 | 25.1 |
| 28/04/2014 | 10.3 | 29.2 |
| 29/04/2014 | 13.0 | 27.1 |
| 30/04/2014 | 12.4 | 26.5 |
| 01/05/2014 | 11.9 | 29.3 |
| 02/05/2014 | 13.4 | 25.2 |
| 03/05/2014 | 12.8 | 35.6 |
| 04/05/2014 | 15.0 | 39.0 |
| 05/05/2014 | 19.1 | 31.5 |
| 06/05/2014 | 18.4 | 29.0 |
| 07/05/2014 | 17.6 | 24.9 |
| 08/05/2014 | 16.6 | 22.9 |
| 09/05/2014 | 14.7 | 25.8 |
| 10/05/2014 | 13.3 | 28.6 |
| 11/05/2014 | 15.8 | 25.7 |
| 12/05/2014 | 13.7 | 25.3 |
| 13/05/2014 | 16.0 | 25.3 |
| 14/05/2014 | 13.8 | 26.1 |
| 15/05/2014 | 11.5 | 29.3 |
| 16/05/2014 | 12.7 | 28.3 |
| 17/05/2014 | 15.0 | 26.4 |
| 18/05/2014 | 15.6 | 25.4 |
| 19/05/2014 | 14.1 | 25.6 |
| 20/05/2014 | 15.6 | 26.5 |
| 21/05/2014 | 15.7 | 32.6 |
| 22/05/2014 | 16.8 | 27.1 |
| 23/05/2014 | 16.7 | 27.5 |
| 24/05/2014 | 14.3 | 27.5 |
| 25/05/2014 | 15.0 | 27.1 |
| 26/05/2014 | 14.5 | 28.1 |
| 27/05/2014 | 16.1 | 32.6 |
| 28/05/2014 | 19.1 | 27.0 |
| 29/05/2014 | 18.1 | 29.0 |
| 30/05/2014 | 14.9 | 35.3 |
| 31/05/2014 | 19.1 | 31.4 |
| 01/06/2014 | 16.6 | 27.3 |
| 02/06/2014 | 17.0 | 27.6 |
| 03/06/2014 | 15.3 | 30.7 |
| 04/06/2014 | 18.8 | 40.7 |
| 05/06/2014 | 20.5 | 28.5 |
| 06/06/2014 | 18.8 | 27.9 |
| 07/06/2014 | 17.2 | 28.6 |
| 08/06/2014 | 16.9 | 28.7 |
| 09/06/2014 | 16.3 | 27.8 |
| 10/06/2014 | 16.9 | 28.5 |
| 11/06/2014 | 17.9 | 28.7 |
| 12/06/2014 | 16.3 | 29.0 |
| 13/06/2014 | 18.1 | 29.5 |
| 14/06/2014 | 17.8 | 30.5 |
| 15/06/2014 | 16.4 | 31.8 |
| 16/06/2014 | 15.2 | 31.9 |
| 17/06/2014 | 18.2 | 31.4 |
| 18/06/2014 | 18.7 | 29.9 |
| 19/06/2014 | 20.3 | 31.5 |
| 20/06/2014 | 22.1 | 36.0 |
| 21/06/2014 | 22.2 | 30.2 |
| 22/06/2014 | 18.7 | 30.5 |
| 23/06/2014 | 18.5 | 29.7 |
| 24/06/2014 | 17.7 | 30.6 |
| 25/06/2014 | 19.0 | 31.1 |
| 26/06/2014 | 18.6 | 31.6 |
| 27/06/2014 | 17.8 | 32.5 |
| 28/06/2014 | 18.9 | 34.2 |
| 29/06/2014 | 20.0 | 34.0 |
| 30/06/2014 | 21.4 | 32.2 |f
f. Near ambient temperatures during the growth season of the 2012 BC2F2 population for marker assisted mapping (Table 2b) and yield parameters analysis (Figure 4). Seedlings were planted in the net house 24.2.2014 and analyzed for yielding on 10-11.6.2014.

## Slide 4
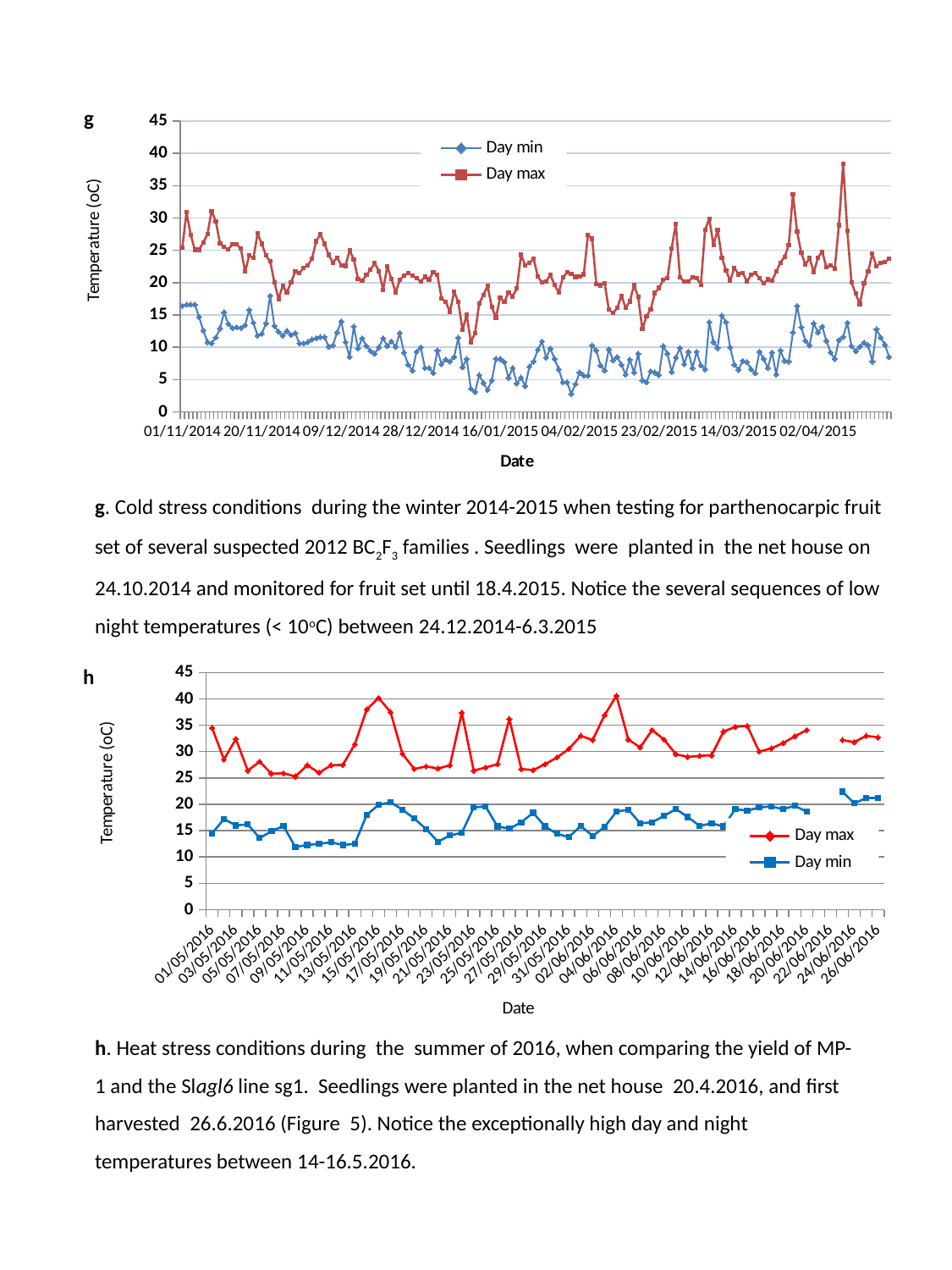

g
### Chart
| Category | Day min | Day max |
|---|---|---|
| 01/11/2014 | 16.4 | 25.4 |
| 02/11/2014 | 16.6 | 30.9 |
| 03/11/2014 | 16.6 | 27.4 |
| 04/11/2014 | 16.6 | 25.1 |
| 05/11/2014 | 14.7 | 25.1 |
| 06/11/2014 | 12.6 | 26.2 |
| 07/11/2014 | 10.8 | 27.5 |
| 08/11/2014 | 10.6 | 31.1 |
| 09/11/2014 | 11.5 | 29.5 |
| 10/11/2014 | 12.9 | 26.1 |
| 11/11/2014 | 15.4 | 25.5 |
| 12/11/2014 | 13.6 | 25.2 |
| 13/11/2014 | 13.0 | 25.9 |
| 14/11/2014 | 13.1 | 25.9 |
| 15/11/2014 | 13.0 | 25.3 |
| 16/11/2014 | 13.4 | 21.7 |
| 17/11/2014 | 15.8 | 24.2 |
| 18/11/2014 | 13.8 | 23.8 |
| 19/11/2014 | 11.8 | 27.6 |
| 20/11/2014 | 12.1 | 26.0 |
| 21/11/2014 | 13.7 | 24.2 |
| 22/11/2014 | 18.0 | 23.3 |
| 23/11/2014 | 13.3 | 20.1 |
| 24/11/2014 | 12.4 | 17.5 |
| 25/11/2014 | 11.8 | 19.5 |
| 26/11/2014 | 12.5 | 18.5 |
| 27/11/2014 | 11.9 | 20.1 |
| 28/11/2014 | 12.2 | 21.7 |
| 29/11/2014 | 10.6 | 21.5 |
| 30/11/2014 | 10.6 | 22.3 |
| 01/12/2014 | 10.8 | 22.7 |
| 02/12/2014 | 11.2 | 23.7 |
| 03/12/2014 | 11.4 | 26.4 |
| 04/12/2014 | 11.6 | 27.5 |
| 05/12/2014 | 11.6 | 26.0 |
| 06/12/2014 | 10.1 | 24.3 |
| 07/12/2014 | 10.3 | 23.1 |
| 08/12/2014 | 12.3 | 23.9 |
| 09/12/2014 | 14.0 | 22.7 |
| 10/12/2014 | 10.8 | 22.6 |
| 11/12/2014 | 8.5 | 25.0 |
| 12/12/2014 | 13.2 | 23.6 |
| 13/12/2014 | 9.8 | 20.6 |
| 14/12/2014 | 11.4 | 20.3 |
| 15/12/2014 | 10.2 | 21.2 |
| 16/12/2014 | 9.4 | 22.0 |
| 17/12/2014 | 9.0 | 23.0 |
| 18/12/2014 | 10.0 | 21.8 |
| 19/12/2014 | 11.4 | 18.9 |
| 20/12/2014 | 10.2 | 22.5 |
| 21/12/2014 | 10.9 | 20.6 |
| 22/12/2014 | 10.1 | 18.5 |
| 23/12/2014 | 12.2 | 20.4 |
| 24/12/2014 | 9.2 | 21.1 |
| 25/12/2014 | 7.3 | 21.5 |
| 26/12/2014 | 6.4 | 21.1 |
| 27/12/2014 | 9.3 | 20.7 |
| 28/12/2014 | 10.0 | 20.2 |
| 29/12/2014 | 6.8 | 20.9 |
| 30/12/2014 | 6.8 | 20.5 |
| 31/12/2014 | 6.0 | 21.6 |
| 01/01/2015 | 9.5 | 21.2 |
| 02/01/2015 | 7.4 | 17.6 |
| 03/01/2015 | 8.1 | 17.0 |
| 04/01/2015 | 7.8 | 15.5 |
| 05/01/2015 | 8.5 | 18.6 |
| 06/01/2015 | 11.5 | 17.0 |
| 07/01/2015 | 6.9 | 12.7 |
| 08/01/2015 | 8.2 | 15.0 |
| 09/01/2015 | 3.6 | 10.8 |
| 10/01/2015 | 3.1 | 12.2 |
| 11/01/2015 | 5.7 | 16.8 |
| 12/01/2015 | 4.5 | 18.1 |
| 13/01/2015 | 3.4 | 19.5 |
| 14/01/2015 | 4.9 | 16.2 |
| 15/01/2015 | 8.2 | 14.6 |
| 16/01/2015 | 8.2 | 17.7 |
| 17/01/2015 | 7.7 | 17.0 |
| 18/01/2015 | 5.3 | 18.5 |
| 19/01/2015 | 6.8 | 17.9 |
| 20/01/2015 | 4.4 | 19.1 |
| 21/01/2015 | 5.3 | 24.3 |
| 22/01/2015 | 4.0 | 22.7 |
| 23/01/2015 | 7.0 | 23.1 |
| 24/01/2015 | 7.8 | 23.7 |
| 25/01/2015 | 9.6 | 21.0 |
| 26/01/2015 | 10.9 | 20.0 |
| 27/01/2015 | 8.4 | 20.2 |
| 28/01/2015 | 9.8 | 21.2 |
| 29/01/2015 | 8.2 | 19.7 |
| 30/01/2015 | 6.6 | 18.5 |
| 31/01/2015 | 4.6 | 20.8 |
| 01/02/2015 | 4.6 | 21.6 |
| 02/02/2015 | 2.8 | 21.4 |
| 03/02/2015 | 4.3 | 20.9 |
| 04/02/2015 | 6.1 | 21.0 |
| 05/02/2015 | 5.6 | 21.3 |
| 06/02/2015 | 5.6 | 27.4 |
| 07/02/2015 | 10.3 | 26.8 |
| 08/02/2015 | 9.5 | 19.8 |
| 09/02/2015 | 7.2 | 19.6 |
| 10/02/2015 | 6.4 | 19.9 |
| 11/02/2015 | 9.7 | 15.8 |
| 12/02/2015 | 8.0 | 15.3 |
| 13/02/2015 | 8.5 | 16.1 |
| 14/02/2015 | 7.3 | 18.0 |
| 15/02/2015 | 5.8 | 16.1 |
| 16/02/2015 | 8.1 | 17.1 |
| 17/02/2015 | 6.1 | 19.6 |
| 18/02/2015 | 9.0 | 17.8 |
| 19/02/2015 | 4.9 | 12.8 |
| 20/02/2015 | 4.6 | 14.8 |
| 21/02/2015 | 6.3 | 15.9 |
| 22/02/2015 | 6.1 | 18.4 |
| 23/02/2015 | 5.7 | 19.2 |
| 24/02/2015 | 10.2 | 20.4 |
| 25/02/2015 | 9.0 | 20.7 |
| 26/02/2015 | 6.2 | 25.3 |
| 27/02/2015 | 8.4 | 29.1 |
| 28/02/2015 | 9.9 | 20.8 |
| 01/03/2015 | 7.4 | 20.2 |
| 02/03/2015 | 9.3 | 20.2 |
| 03/03/2015 | 6.8 | 20.8 |
| 04/03/2015 | 9.3 | 20.7 |
| 05/03/2015 | 7.2 | 19.7 |
| 06/03/2015 | 6.6 | 28.2 |
| 07/03/2015 | 13.9 | 29.9 |
| 08/03/2015 | 10.8 | 25.8 |
| 09/03/2015 | 9.9 | 28.2 |
| 10/03/2015 | 14.9 | 23.9 |
| 11/03/2015 | 13.9 | 21.9 |
| 12/03/2015 | 10.0 | 20.3 |
| 13/03/2015 | 7.3 | 22.3 |
| 14/03/2015 | 6.5 | 21.3 |
| 15/03/2015 | 7.9 | 21.5 |
| 16/03/2015 | 7.7 | 20.2 |
| 17/03/2015 | 6.6 | 21.2 |
| 18/03/2015 | 6.0 | 21.5 |
| 19/03/2015 | 9.3 | 20.7 |
| 20/03/2015 | 8.2 | 19.9 |
| 21/03/2015 | 6.8 | 20.5 |
| 22/03/2015 | 9.2 | 20.3 |
| 23/03/2015 | 5.8 | 21.7 |
| 24/03/2015 | 9.5 | 23.1 |
| 25/03/2015 | 7.9 | 24.0 |
| 26/03/2015 | 7.8 | 25.8 |
| 27/03/2015 | 12.3 | 33.7 |
| 28/03/2015 | 16.4 | 27.9 |
| 29/03/2015 | 13.1 | 24.6 |
| 30/03/2015 | 11.0 | 22.8 |
| 31/03/2015 | 10.3 | 23.8 |
| 01/04/2015 | 13.7 | 21.6 |
| 02/04/2015 | 12.3 | 23.9 |
| 03/04/2015 | 13.2 | 24.8 |
| 04/04/2015 | 11.0 | 22.4 |
| 05/04/2015 | 9.2 | 22.7 |
| 06/04/2015 | 8.2 | 22.2 |
| 07/04/2015 | 11.1 | 28.9 |
| 08/04/2015 | 11.6 | 38.4 |
| 09/04/2015 | 13.8 | 28.0 |
| 10/04/2015 | 10.2 | 20.1 |
| 11/04/2015 | 9.4 | 18.3 |
| 12/04/2015 | 10.1 | 16.7 |
| 13/04/2015 | 10.7 | 19.9 |
| 14/04/2015 | 10.3 | 21.8 |
| 15/04/2015 | 7.8 | 24.5 |
| 16/04/2015 | 12.8 | 22.5 |
| 17/04/2015 | 11.5 | 23.1 |
| 18/04/2015 | 10.4 | 23.2 |
| 19/04/2015 | 8.5 | 23.7 |g. Cold stress conditions during the winter 2014-2015 when testing for parthenocarpic fruit set of several suspected 2012 BC2F3 families . Seedlings were planted in the net house on 24.10.2014 and monitored for fruit set until 18.4.2015. Notice the several sequences of low night temperatures (< 10oC) between 24.12.2014-6.3.2015
### Chart
| Category | Day max | Day min |
|---|---|---|
| 01/05/2016 | 34.5 | 14.5 |
| 02/05/2016 | 28.5 | 17.2 |
| 03/05/2016 | 32.4 | 16.0 |
| 04/05/2016 | 26.4 | 16.2 |
| 05/05/2016 | 28.1 | 13.6 |
| 06/05/2016 | 25.8 | 14.9 |
| 07/05/2016 | 25.9 | 15.9 |
| 08/05/2016 | 25.3 | 11.9 |
| 09/05/2016 | 27.4 | 12.3 |
| 10/05/2016 | 26.0 | 12.5 |
| 11/05/2016 | 27.4 | 12.8 |
| 12/05/2016 | 27.5 | 12.3 |
| 13/05/2016 | 31.4 | 12.5 |
| 14/05/2016 | 38.0 | 18.0 |
| 15/05/2016 | 40.2 | 19.9 |
| 16/05/2016 | 37.5 | 20.4 |
| 17/05/2016 | 29.6 | 19.0 |
| 18/05/2016 | 26.7 | 17.3 |
| 19/05/2016 | 27.2 | 15.3 |
| 20/05/2016 | 26.8 | 12.9 |
| 21/05/2016 | 27.4 | 14.1 |
| 22/05/2016 | 37.4 | 14.6 |
| 23/05/2016 | 26.4 | 19.4 |
| 24/05/2016 | 27.0 | 19.6 |
| 25/05/2016 | 27.6 | 15.8 |
| 26/05/2016 | 36.2 | 15.4 |
| 27/05/2016 | 26.7 | 16.6 |
| 28/05/2016 | 26.5 | 18.4 |
| 29/05/2016 | 27.6 | 15.8 |
| 30/05/2016 | 28.9 | 14.4 |
| 31/05/2016 | 30.5 | 13.8 |
| 01/06/2016 | 33.0 | 15.9 |
| 02/06/2016 | 32.2 | 14.0 |
| 03/06/2016 | 36.9 | 15.7 |
| 04/06/2016 | 40.6 | 18.6 |
| 05/06/2016 | 32.3 | 19.0 |
| 06/06/2016 | 30.8 | 16.4 |
| 07/06/2016 | 34.1 | 16.6 |
| 08/06/2016 | 32.3 | 17.8 |
| 09/06/2016 | 29.5 | 19.1 |
| 10/06/2016 | 29.0 | 17.6 |
| 11/06/2016 | 29.2 | 15.9 |
| 12/06/2016 | 29.3 | 16.4 |
| 13/06/2016 | 33.8 | 15.8 |
| 14/06/2016 | 34.7 | 19.1 |
| 15/06/2016 | 34.9 | 18.8 |
| 16/06/2016 | 30.0 | 19.4 |
| 17/06/2016 | 30.6 | 19.6 |
| 18/06/2016 | 31.6 | 19.1 |
| 19/06/2016 | 32.9 | 19.7 |
| 20/06/2016 | 34.1 | 18.6 |
| 21/06/2016 | None | None |
| 22/06/2016 | None | None |
| 23/06/2016 | 32.2 | 22.4 |
| 24/06/2016 | 31.8 | 20.2 |
| 25/06/2016 | 33.0 | 21.2 |
| 26/06/2016 | 32.7 | 21.2 |h
h. Heat stress conditions during the summer of 2016, when comparing the yield of MP-1 and the Slagl6 line sg1. Seedlings were planted in the net house 20.4.2016, and first harvested 26.6.2016 (Figure 5). Notice the exceptionally high day and night temperatures between 14-16.5.2016.
