# Supplementary material for: Tomato facultative parthenocarpy results from Sl AGAMOUS‐LIKE 6 loss of function
Source: Plant Biotechnol J. 2016 Dec 27;15(5):634–47. doi: 10.1111/pbi.12662 (PMC5399002; doi:10.1111/pbi.12662)
Supplement: Supplementary file 4 — Figure S4. Reported modes of expression of SlAGL6. [file PBI-15-634-s001.pptx]

## Slide 1
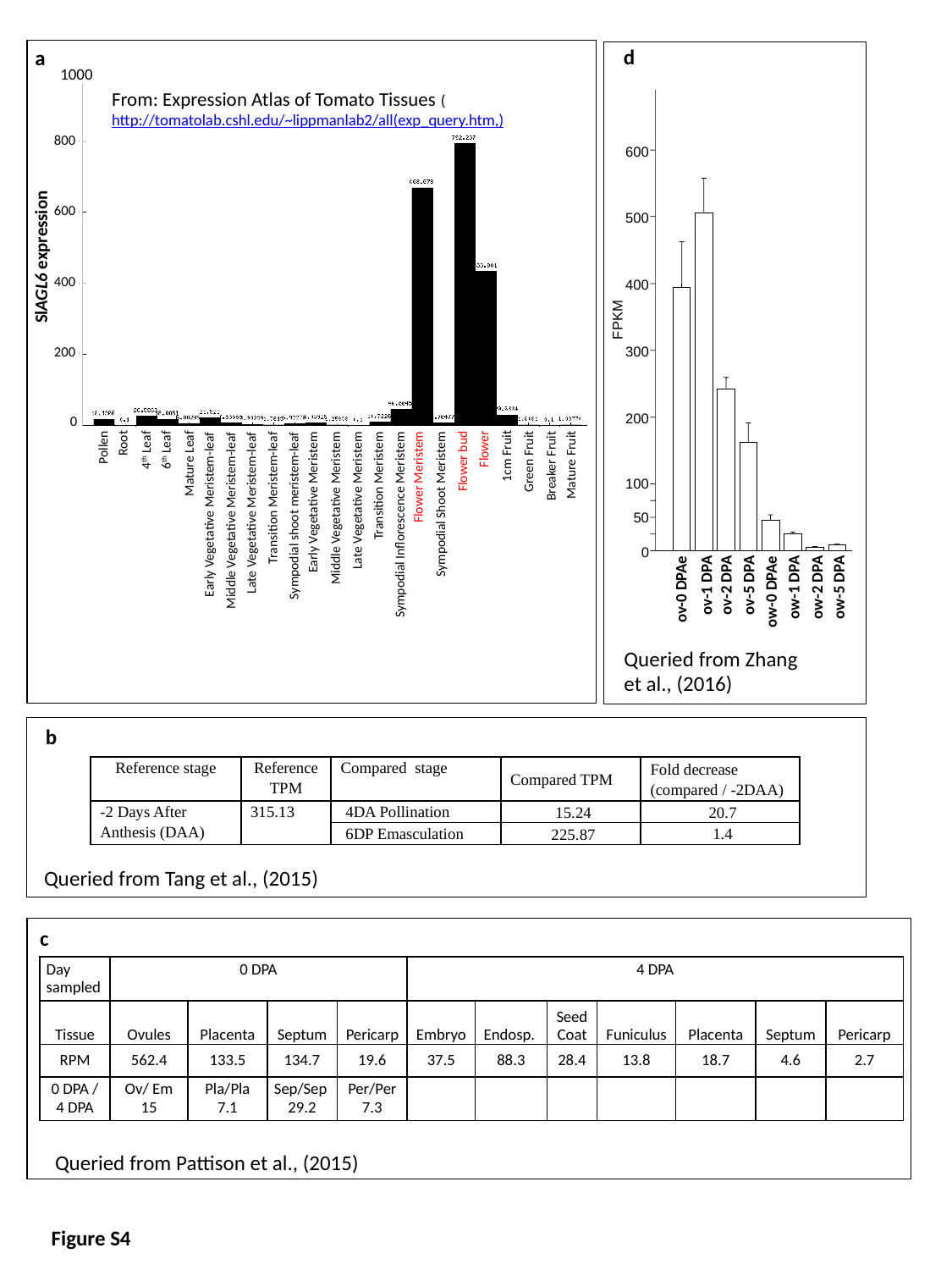

d
a
1000
From: Expression Atlas of Tomato Tissues (http://tomatolab.cshl.edu/~lippmanlab2/all(exp_query.htm,)
800
600
600
500
SlAGL6 expression
400
400
FPKM
200
300
200
0
Root
Pollen
Flower
4th Leaf
6th Leaf
1cm Fruit
Flower bud
Green Fruit
Mature Leaf
Mature Fruit
Breaker Fruit
Flower Meristem
100
Transition Meristem
Transition Meristem-leaf
Late Vegetative Meristem
Early Vegetative Meristem
Sympodial Shoot Meristem
Middle Vegetative Meristem
Late Vegetative Meristem-leaf
Early Vegetative Meristem-leaf
Sympodial shoot meristem-leaf
50
Middle Vegetative Meristem-leaf
Sympodial Inflorescence Meristem
0
ov-2 DPA
ov-5 DPA
ov-1 DPA
ow-1 DPA
ow-2 DPA
ow-5 DPA
ov-0 DPAe
ow-0 DPAe
Queried from Zhang et al., (2016)
b
| Reference stage | Reference TPM | Compared stage | Compared TPM | Fold decrease (compared / -2DAA) |
| --- | --- | --- | --- | --- |
| -2 Days After Anthesis (DAA) | 315.13 | 4DA Pollination | 15.24 | 20.7 |
| | | 6DP Emasculation | 225.87 | 1.4 |
Queried from Tang et al., (2015)
c
| Day sampled | 0 DPA | | | | 4 DPA | | | | | | |
| --- | --- | --- | --- | --- | --- | --- | --- | --- | --- | --- | --- |
| Tissue | Ovules | Placenta | Septum | Pericarp | Embryo | Endosp. | Seed Coat | Funiculus | Placenta | Septum | Pericarp |
| RPM | 562.4 | 133.5 | 134.7 | 19.6 | 37.5 | 88.3 | 28.4 | 13.8 | 18.7 | 4.6 | 2.7 |
| 0 DPA / 4 DPA | Ov/ Em 15 | Pla/Pla 7.1 | Sep/Sep 29.2 | Per/Per 7.3 | | | | | | | |
Queried from Pattison et al., (2015)
Figure S4

## Slide 2
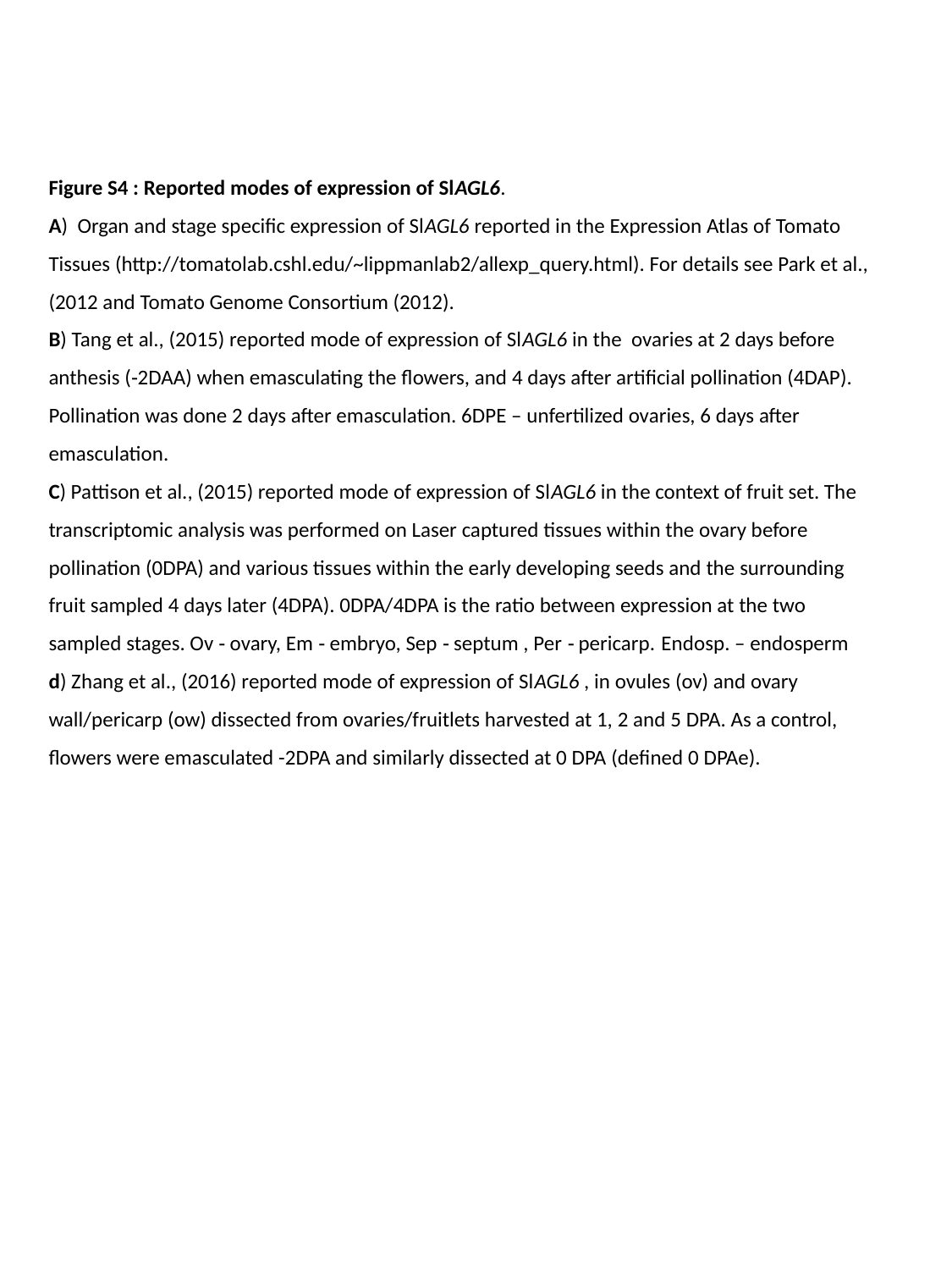

Figure S4 : Reported modes of expression of SlAGL6.
A) Organ and stage specific expression of SlAGL6 reported in the Expression Atlas of Tomato Tissues (http://tomatolab.cshl.edu/~lippmanlab2/allexp_query.html). For details see Park et al., (2012 and Tomato Genome Consortium (2012).
B) Tang et al., (2015) reported mode of expression of SlAGL6 in the ovaries at 2 days before anthesis (‐2DAA) when emasculating the flowers, and 4 days after artificial pollination (4DAP). Pollination was done 2 days after emasculation. 6DPE – unfertilized ovaries, 6 days after emasculation.
C) Pattison et al., (2015) reported mode of expression of SlAGL6 in the context of fruit set. The transcriptomic analysis was performed on Laser captured tissues within the ovary before pollination (0DPA) and various tissues within the early developing seeds and the surrounding fruit sampled 4 days later (4DPA). 0DPA/4DPA is the ratio between expression at the two sampled stages. Ov ‐ ovary, Em ‐ embryo, Sep ‐ septum , Per ‐ pericarp. Endosp. – endosperm
d) Zhang et al., (2016) reported mode of expression of SlAGL6 , in ovules (ov) and ovary wall/pericarp (ow) dissected from ovaries/fruitlets harvested at 1, 2 and 5 DPA. As a control, flowers were emasculated -2DPA and similarly dissected at 0 DPA (defined 0 DPAe).
