# Supplementary material for: Tomato facultative parthenocarpy results from Sl AGAMOUS‐LIKE 6 loss of function
Source: Plant Biotechnol J. 2016 Dec 27;15(5):634–47. doi: 10.1111/pbi.12662 (PMC5399002; doi:10.1111/pbi.12662)
Supplement: Supplementary file 5 — Table S1. Primers used in this study. [file PBI-15-634-s002.docx]

**Table S1:** List of used primers

| Experiment | Name | Sequence 5' - 3' |
| --- | --- | --- |
| qRT-PCR analysis | SlTIP41-F | ATGGAGTTTTTGAGTCTTCTGC |
|  | SlTIP41-R | GCTGCGTTTCTGGCTTAGG |
|  | SlAGL6-F | AAACCCTTGAGAGGTACCAACG |
|  | SlAGL6-R | CACCAAGCAAGTGCCTTTGAG |
| Cas9 editing detection | 2012-F | GCCTTGAAATCAGTAAGAGTATTGG |
|  | 2012-R | GTTCGTTGAAGTGCTTCAAACTTGG |
| Cas9 cassette detection | cas-F | CGACAATCTGATCCAAGCTCA |
|  | cas-R | GACACTGACGGCTTTATGCC |
