# Supplementary material for: Tomato facultative parthenocarpy results from Sl AGAMOUS‐LIKE 6 loss of function
Source: Plant Biotechnol J. 2016 Dec 27;15(5):634–47. doi: 10.1111/pbi.12662 (PMC5399002; doi:10.1111/pbi.12662)
Supplement: Supplementary file 6 — Data S1. Methods. [file PBI-15-634-s003.docx]

**Data S1**

**Supplementary Methods**

**Screening the EMS induced M_2_ population for yielding under extreme temperatures:** A chemically (EMS) mutagenized M_2_ population generated in the M82 cultivar (generated by J. Hirschenhoren and Y. Kapulnik, The Volcani Center, ARO), was screened for mutants yielding under extremely high temperatures (Figure S3a), which prevented fertilization-dependent fruit set (Sato et al., 2006). Twelve plants from each of 1000 M_2_ EMS mutagenized families generated in the M82 background were planted in the field in the summer. The field was re-screened for fruit set during the very hot months of August-September. Single plants from several families set parthenocarpic fruits, yet except for the chosen mutation 2012, their seedless fruits were either distorted or relatively small (< 35g), presumably bearing mutations which only partly mimic fertilization-dependent fruit development.

**Generation of populations for mapping the 2012 mutant, growth conditions, and phenotyping:**

**2012 BC_1_F_2_ population:** The BC_1_ was generated by pollinating emasculated flowers of line M82 with pollen collected from the 2012 plant. These plants were grown and allowed to self to generate BC_1_F_2_. The BC_1_F_2_ population served to generate the two genomic libraries subjected to deep sequencing.

From the 2012 BC_1_F_2_ population, 105 plants were grown in the late summer, since from mid-July to late August the temperatures were high enough (Figure S3d) to seriously damage microsporogenesis (Sato et al., 2006) and hence fertilization-dependent fruit development (Figure 1a *vs.*1b). In the late summer the fruits were harvested examined, and each plant was phenotyped. The parthenocarpic phenotype was determined based on yielding nice parthenocarpic fruits with good jelly fill (e.g. Figure 1a). Sibling plants were characterized as non-parthenocarpic (NP) if no seedless normal size fruits developed (similar to Figure 1a,b).

**Test-cross (TC) population:** Several 2012 BC_1_F_2_ plants manifesting WT phenotype, i.e. not parthenocarpic were emasculated and pollinated with pollen collected from a parthenocarpic sibling. About 100 progenies from each of three of these crosses were planted in the summer. One of the F_1_ populations was identified as a TC population since it manifested a clear 1:1 segregation for the parthenocarpic phenotype, when yielding under the high temperatures prevailing during the late summer in the net-house (Figure S3e). This population served for the co-segregation analysis summarized in Table 2

**2012 BC_2_F_2_ population:** One of the plants from the BC_1_F_2_ population showing a clear parthenocarpic phenotype was used to pollinate the parental line M82, the F_1_ plants were grown and selfed to generate BC_2_F_2_. Unlike the BC_1_F_2_ and the TC populations, that were phenotyped under extremely high temperatures, to hasten the analysis, the BC_2_F_2_ population was tested in the spring of 2014, under near-ambient temperatures for fertilization (Figure 3Sf). Towards the end of the winter (26.2.2014), 498 BC_2_F_2_ plants were planted in a non-heated net-house, together with plants from the parental line M82. At the beginning of May 2014, some of the BC_2_F_2_ plants were already bearing red and even red-ripe fruits. Hence the first round of phenotyping was performed on the 13-15 of May 2014. Since the 2012 mutation was found to cause a strong yet facultative parthenocarpy, a stringent protocol was set for phenotyping the progenies, especially as they grew and set fruit under nearly ambient conditions (Figure S3f). From each plant the 6-8 most red ripen fruits were picked. If none were that ripen, as in the control M82 cultivar and most of the BC_2_F2 plants, the six largest green fruits were picked. These were photographed, cut transversely and the presence or absence of seeds was recorded. Plants bearing big seedless usually red fruits were defined as parthenocarpic, even if some of the picked fruits contained few seeds. Plants were defined as non-parthenocarpic if delayed in fruit development, and most importantly, setting only seeded fruits, even if the number of seeds per some of their fruits was low. For the analysis of red fruit yield parameters under ambient growth conditions, (Fig. 4a-c), for each Sl*AGL6* genotype (SNP No. 3), the number of plants specified in Figure 1a-c were harvested on 10 of June 2014 and the weight and number of red fruits was recorded. To reduce environmental effects on yielding, plants located at the ends of the rows were not included in the analysis.

**Bioinformatics analysis of the sequenced 2012 and NP (non-parthenocarpic) genomic libraries:**

The raw sequence data, comprising 15.4Gbp and 15.9 GBp for the 2012 and the NP pools, respectively, was filtered using Trimmomatic (version V0.30) to remove adapter and low quality sequence (below Q10) (Bolger et al., 2014b). The resulting datasets were aligned using BWA (Li and Durbin 2009) against the M82 sequence (Bolger et al., 2014a). The alignments were filtered to remove ambiguous, secondary, or pairwise discordant alignments. The remaining high quality alignments where then used with SAMtools (Li et al., 2009) to generate a ‘read pileup’ for the parthenocarpic and non-parthenocarpic pools. Custom scripts were used to interpret the pileup and identify genomic locations where the pools differed substantially, following the approach of SHOREmap (Schneeberger et al., 2009). These regions were then plotted and used to identify a 10.4Mbp region on chromosome one, from 75.8Mbp to 86.2Mbp, which most likely contained the causal mutation. It was possible to identify 19 high-confidence mutations within this window which were unique to the parthenocarpic pool. The sequence around each mutation was extracted and mapped using BLAST to the Heinz genome. The majority of the mutations were intergenic or in introns. Of the total of 4 which hit exons, 2 were synonymous changes and 2 were non-synonymous changes with minor effect (Valine to Isoleucine). In addition, the functional annotation of the affected genes did not suggest an obvious connection to the observed phenotype.

To improve the analysis, another lane was sequenced from each of the two libraries. Furthermore, to ensure background differences between the published M82 and local M82 lines did not affect results, the parental M82 line was also sequenced. These datasets comprised 39.6Gbp, 38.1Gp and 31.5Gbp of data for the 2012 library the NP library and the parental M82 line, respectively. All datasets were trimmed using Trimmomatic as before and the 2012 and NP pools were combined with the previously sequenced data. Reads from the parental line were aligned using BWA and almost 30K high-confidence variants were called using SAMtools. These variants were applied to the public M82 sequence to create the parental M82 genome sequence.

The new and existing parthenocarpic and non-parthenocarpic datasets were then aligned using BWA against the newly determined parental M82 genome, and processed as before to determine genomic locations where the pool differed substantially. The larger dataset allowed the causal window to be narrowed to a region spanning from 76.67Mbp to 80.73Mp on chromosome 1, which corresponds to a region spanning from 84.9Mbp to 89.0Mbp on the Heinz genome. Nine high confidence mutations, unique to the 2012 parthenocarpic pool were identified within the window. These were mapped as before to the Heinz genome to determine the corresponding locations, as shown in Table 1. Surprisingly, the strongest candidate mutation from this analysis, which caused an early stop codon in Solyc01g093960, was not clearly detectable in the original dataset, since it was present at 57% (8 of 14 reads) in the non-parthenocarpic pool, far above the 33% expected.

**Identification and genotyping Cas9 generated mutants in R_0_ and R_1_ generation:**

R_o_ plants were screened for the presence of chimeric section carrying mutated target site. To increase the probability of detecting Cas9 generated mutation which occurred at later developmental stages of the regenerated R_0_ plants, genomic DNA was extracted from leaves younger than number 10-12 on the main stem. DNA was amplified using specific primers: 2012-F and 2012-R (Table S1), to result in a 354 bp fragment flanking the sgRNA target sequence. The PCR product was digest with AclI, and subjected to gel electrophoresis. Unless mutated, its digest with AclI results in two bands of similar size (170/184 bp). DNA was extracted from the gel at the position of the uncut band, that even when no clear band was visualized under UV light. The extracted DNA was precipitated over-night at –20^0^C (0.3M Na-Acetate pH=5.2, 2.2V ethanol and 1μg glycogen), re-suspended in 10 μL water, from which 1 μL served as a template for amplification using the same pair of primers. The product obtained was sequenced both with the Forward and Reverse primers.

**The Cas9 derived analyzed mutations**: Based on the above described analysis, the three R_0_ plants which progenies were chosen for the phenotypic analysis (Figure 2b),) did bear the following mutations: **sg1** - a 175 bp deletion, comprising the last 98 bp of intron 1, all of exon 2 (76 bp), and the first bp of intron 2; **sg4** – contained two mutated variants: (1) two nucleotides (G213/T213) deletion , and (2) one nucleotide addition after T213; **sg5** - a single bp (T213) deletion. Their R_1_ progenies were genotyped to determine if they are homozygous (m/m) or heterozygous (+/m) for a mutated allele or homozygous for the WT (+/+) allele of *SlAGL6*, and PCR tested for the presence of the transgene *Cas9* (Table S1). Several of the R_1_ progenies of plants sg4 and sg5 were found to carry new mutated alleles, different from the ones specified above, or being mutated for bi-allelic mutated versions of *SlAGL6*. This is not surprising since they were all found to contain the *Cas9* cassette.

**Analysis of sg1 and MP-1 yielding under heat stress**:

The experiment was performed in four replicate, each consisting of 17-27 plants per genotype. Plants were planted in a net-house on 20 of April 2016 and the first harvest was performed on the 26 of June 2016 (presented in Figure 5, for climatic conditions see Figure S3h). All the red fruits of each replicate were harvested, and weighed. Fruit weight and fruit number per plant were calculated from the weight of 6-8 batches of 30 fruits each per replicate. The data presented in Figure 5a,b was calculate per plant, since the plants' number varied among replicates. Brix was measured on juice squeezed from two pools of three red ripe fruits, per replicate (following Carmi et al., 2003). pH was measured on two pools of crashed 6-8 red ripe fruits per replicate.

**References**

Bolger, A., Scossa, F., Bolger, M.E., Lanz, C., et al. (2014a) The genome of the stress-tolerant wild tomato species Solanum pennellii. *Nat. Genet*. **46**, 1034-1038.

Bolger, A.M., Lohse, M., and Usadel, B. (2014b) Trimmomatic: a flexible trimmer for Illumina sequence data. *Bioinformatics,*  **30**, 2114-2120.

Carmi, N., Salts, Y., Dedicova, B., Shabtai, S. and Barg, R. (2003) Induction of parthenocarpy in tomato via specific expression of the *rolB* gene in the ovary. *Planta*, **217**, 726-735.

Li, H., and Durbin, R. (2009) Fast and accurate short read alignment with Burrows-Wheeler transform. *Bioinformatics,* **25**, 1754-1760.

Li, H., Handsaker, B., Wysoker, A., Fennell, T., Ruan, J. Homer, N., Marth, G., Abecasis, G., Durbin, R. and 1000 Genome Project Data Processing Subgroup (2009) The Sequence Alignment/Map format and SAMtools. *Bioinformatics*, **25**, 2078-2079.

Li, J.F., Norville, J.E., Aach, J., McCormack, M., Zhang, D., Bush, J., Church, G.M., and Sheen, J. (2013). Multiplex and homologous recombination-mediated genome editing in *Arabidopsis* and *Nicotiana benthamiana* using guide RNA and Cas9. *Nat. Biotechnol*. **31**, 688–691.

Sato, S., Kamiyama, M., Iwata, T., Makita, N., Furukawa, H., and Ikeda, H. (2006) Moderate increase of mean daily temperature adversely affects fruit set of Lycopersicon esculentum by disrupting specific physiological processes in male reproductive development. *Ann. Bot.* **97**, 731-738.

Schneeberger, K., Ossowski, S., Lanz, C., Juul, T., Petersen, A.H., Nielsen, K.L., Jørgensen, J.E., Weige, D., and Andersen, S.U. (2009) SHOREmap: simultaneous mapping and mutation identification by deep sequencing. *Nat. Methods*, **6**, 550-551.
